# Supplementary material for: Structures and Bioactivities of Six New Triterpene Glycosides, Psolusosides E, F, G, H, H1, and I and the Corrected Structure of Psolusoside B from the Sea Cucumber Psolus fabricii
Source: Mar Drugs. 2019 Jun 14;17(6):358. doi: 10.3390/md17060358 (PMC6627558; doi:10.3390/md17060358)
Supplement: Supplementary file 1 [file marinedrugs-17-00358-s001.pdf]

Supplementary data content page

Title: Structures and bioactivities of six new triterpene glycosides, psolusosides E, F, G, H, H_1 and I and the corrected structure of psolusoside B from the sea cucumber *Psolus fabricii*

Authors: Alexandra S. Silchenko, Anatoly I. Kalinovsky, Sergey A. Avilov, Vladimir I. Kalinin*, Pelageya V. Andrijaschenko, Pavel S. Dmitrenok, Roman S. Popov, Ekaterina A. Chingizova, Svetlana P. Ermakova, Olesya S. Malyarenko

Address: [1]*G.B. Elyakov Pacific Institute of Bioorganic Chemistry, Far Eastern Branch of Russian Academy of Sciences, Pr. 100-let Vladivostoku 159, 690022 Vladivostok, Russia*

[2]*School of Natural Science, Far Eastern Federal University, Sukhanova St., 8, Vladivostok 690000, Russia*

Correspondence: kalininv@piboc.dvo.ru; Tel.: +7-423-231-1168

Contents:

Fig. 1. The ^{13}C NMR (176.04 MHz) spectrum of psolusoside B (**1**) in in C_5D_5N/D_2O (4/1)

Fig. 2. The ^{1}H NMR (700.13 MHz) spectrum of psolusoside B (**1**) in C_5D_5N/D_2O (4/1)

Fig. 3. The COSY (700.13 MHz) spectrum of psolusoside B (**1**) in C_5D_5N/D_2O (4/1)

Fig. 4. The HSQC (700.13 MHz) spectrum of psolusoside B (**1**) in C_5D_5N/D_2O (4/1)

Fig. 5. The HMBC (700.13 MHz) spectrum of psolusoside B (**1**) in C_5D_5N/D_2O (4/1)

Fig. 6. The ROESY (500.13 MHz) spectrum of psolusoside B (**1**) in C_5D_5N/D_2O (4/1)

Fig. 7. 1 D TOCSY (700.13 MHz) spectra of psolusoside B (**1**) in C_5D_5N/D_2O (4/1)

Fig. 8. 1 D TOCSY (700.13 MHz) spectra of psolusoside B (**1**) in C_5D_5N/D_2O (4/1)

Fig. 9. The ^{13}C NMR (176.04 MHz) spectrum of psolusoside E (**2**) in in C_5D_5N/D_2O (4/1)

Fig. 10. The ^{1}H NMR (700.13 MHz) spectrum of psolusoside E (**2**) in C_5D_5N/D_2O (4/1)

Fig. 11. The COSY (700.13 MHz) spectrum of psolusoside E (**2**) in C_5D_5N/D_2O (4/1)

Fig. 12. The HSQC (700.13 MHz) spectrum of psolusoside E (**2**) in C_5D_5N/D_2O (4/1)

Fig. 13. The ROESY (500.13 MHz) spectrum of psolusoside E (**2**) in C_5D_5N/D_2O (4/1)

Fig. 14. The HMBC (700.13 MHz) spectrum of psolusoside E (**2**) in C_5D_5N/D_2O (4/1)

Fig. 15. 1D TOCSY (700.13 MHz) spectra of psolusoside E (**2**) in C_5D_5N/D_2O (4/1)

Fig. 16. 1D TOCSY (700.13 MHz) spectra of psolusoside E (**2**) in C_5D_5N/D_2O (4/1)

Fig. 17. The ^{13}C NMR (176.04 MHz) spectrum of psolusoside F (**3**) in in C_5D_5N/D_2O (4/1)

Fig. 18. The ^{1}H NMR (700.13 MHz) spectrum of psolusoside F (**3**) in C_5D_5N/D_2O (4/1)

Fig. 19. The COSY (700.13 MHz) spectrum of psolusoside F (**3**) in C_5D_5N/D_2O (4/1)

Fig. 20. The HSQC (700.13 MHz) spectrum of psolusoside F (**3**) in C_5D_5N/D_2O (4/1)

Fig. 21. The HMBC (700.13 MHz) spectrum of psolusoside F (**3**) in C_5D_5N/D_2O (4/1)

Fig. 22. The ROESY (500.13 MHz) spectrum of psolusoside F (**3**) in C_5D_5N/D_2O (4/1)

Fig. 23. 1D TOCSY (700.13 MHz) spectra of psolusoside F (**3**) in C_5D_5N/D_2O (4/1)

Fig. 24. 1D TOCSY (700.13 MHz) spectra of psolusoside F (**3**) in C_5D_5N/D_2O (4/1)

Fig. 25. The ^{13}C NMR (176.04 MHz) spectrum of psolusoside G (**4**) in in C_5D_5N/D_2O (4/1)

Fig. 26. The ^{1}H NMR (700.13 MHz) spectrum of psolusoside G (**4**) in C_5D_5N/D_2O (4/1)

Fig. 27. The COSY (700.13 MHz) spectrum of psolusoside G (**4**) in C_5D_5N/D_2O (4/1)

Fig. 28. The HSQC (700.13 MHz) spectrum of psolusoside G (**4**) in C_5D_5N/D_2O (4/1)

Fig. 29. The ROESY (500.13 MHz) spectrum of psolusoside G (**4**) in C_5D_5N/D_2O (4/1)

Fig. 30. The HMBC (700.13 MHz) spectrum of psolusoside G (**4**) in C_5D_5N/D_2O (4/1)

Fig. 31. 1D TOCSY (700.13 MHz) spectra of psolusoside G (**4**) in C_5D_5N/D_2O (4/1)

Fig. 32. 1D TOCSY (700.13 MHz) spectra of psolusoside G (**4**) in C_5D_5N/D_2O (4/1)

Fig. 33. The ^{13}C NMR (176.04 MHz) spectrum of psolusoside H (**5**) in C_5D_5N

Fig. 34. The ^{1}H NMR (700.13 MHz) spectrum of psolusoside H (**5**) in C_5D_5N

Fig. 35. The COSY (700.13 MHz) spectrum of psolusoside H (**5**) in C_5D_5N

Fig. 36. The HSQC (700.13 MHz) spectrum of psolusoside H (**5**) in C_5D_5N

Fig. 37. The HMBC (700.13 MHz) spectrum of psolusoside H (**5**) in C_5D_5N

Fig. 38. The ROESY (500.13 MHz) spectrum of psolusoside H (**5**) in C_5D_5N

Fig. 39. 1D TOCSY (700.13 MHz) spectra of psolusosides H (**5**) and H_1 (**6**) in C_5D_5N

Fig. 40. The ^{13}C NMR (176.04 MHz) spectrum of psolusoside H_1 (**6**) in C_5D_5N

Fig. 41. The ^{1}H NMR (700.13 MHz) spectrum of psolusoside H_1 (**6**) in C_5D_5N

Fig. 42. The COSY (700.13 MHz) spectrum of psolusoside H_1 (**6**) in C_5D_5N

Fig. 43. The HSQC (700.13 MHz) spectrum of psolusoside H_1 (**6**) in C_5D_5N

Fig. 44. The HMBC (700.13 MHz) spectrum of psolusoside H_1 (**6**) in C_5D_5N

Fig. 45. The ROESY (500.13 MHz) spectrum of psolusoside H_1 (**6**) in C_5D_5N

Fig. 46. The ^{13}C NMR (176.04 MHz) spectrum of psolusoside I (**7**) in C_5D_5N/D_2O (4/1)

Fig. 47. The ^{1}H NMR (700.13 MHz) spectrum of psolusoside I (**7**) in C_5D_5N/D_2O (4/1)

Fig. 49. The COSY (700.13 MHz) spectrum of psolusoside I (**7**) in C_5D_5N/D_2O (4/1)

Fig. 50. The HSQC (700.13 MHz) spectrum of psolusoside I (**7**) in C_5D_5N/D_2O (4/1)

Fig. 51. The HMBC (700.13 MHz) spectrum of psolusoside I (**7**) in C_5D_5N/D_2O (4/1)

Fig. 52. The ROESY (500.13 MHz) spectrum of psolusoside I (**7**) in C_5D_5N/D_2O (4/1)

Fig. 53. 1D TOCSY (700.13 MHz) spectra of psolusoside I (**7**) in C_5D_5N/D_2O (4/1)

Fig. 54. 1D TOCSY (700.13 MHz) spectra of psolusoside I (**7**) in C_5D_5N/D_2O (4/1)

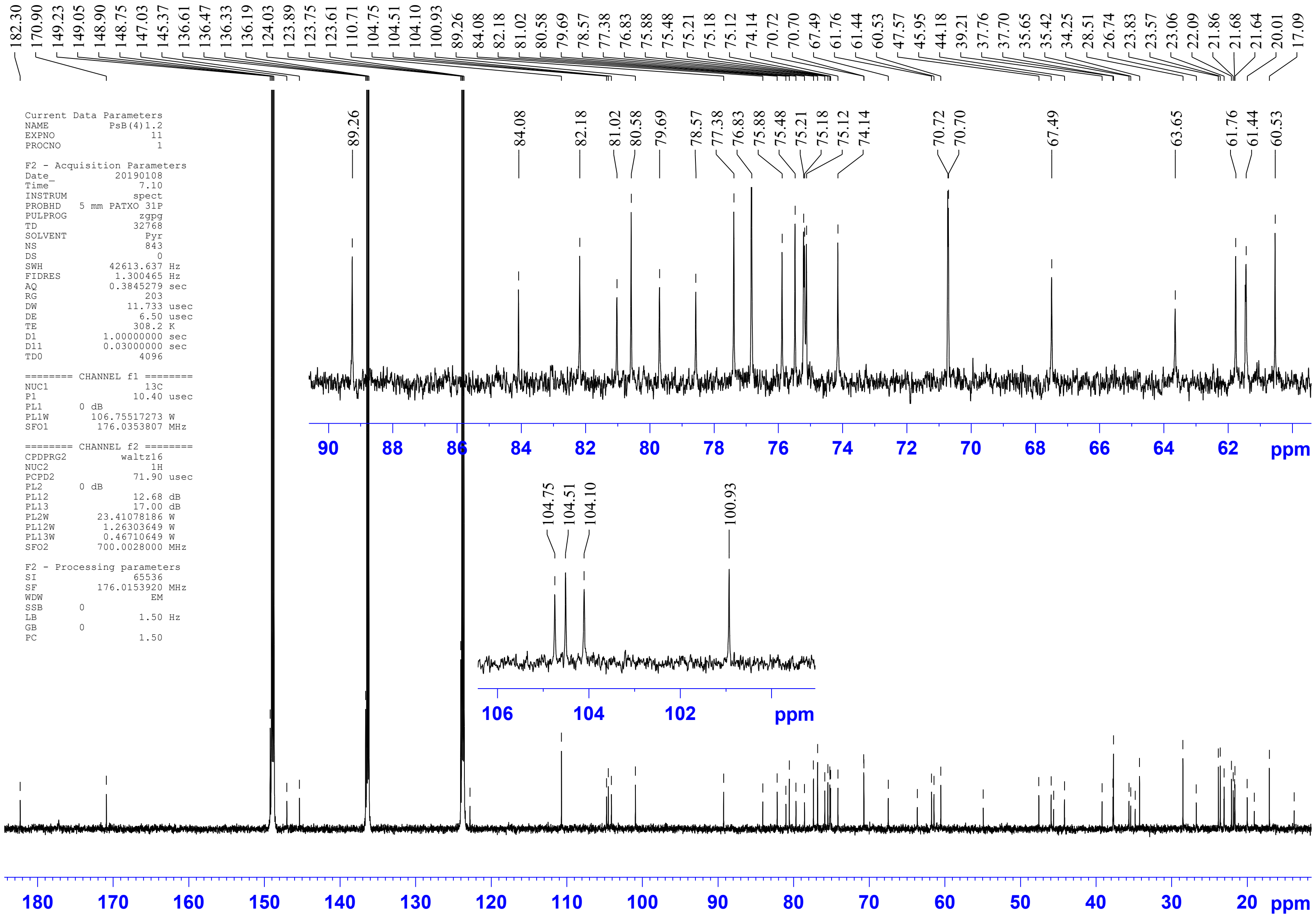

Fig. 1. The ^{13}C NMR (176.04 MHz) spectrum of psolusoside B (**1**) in in C_5D_5N/D_2O (4/1)

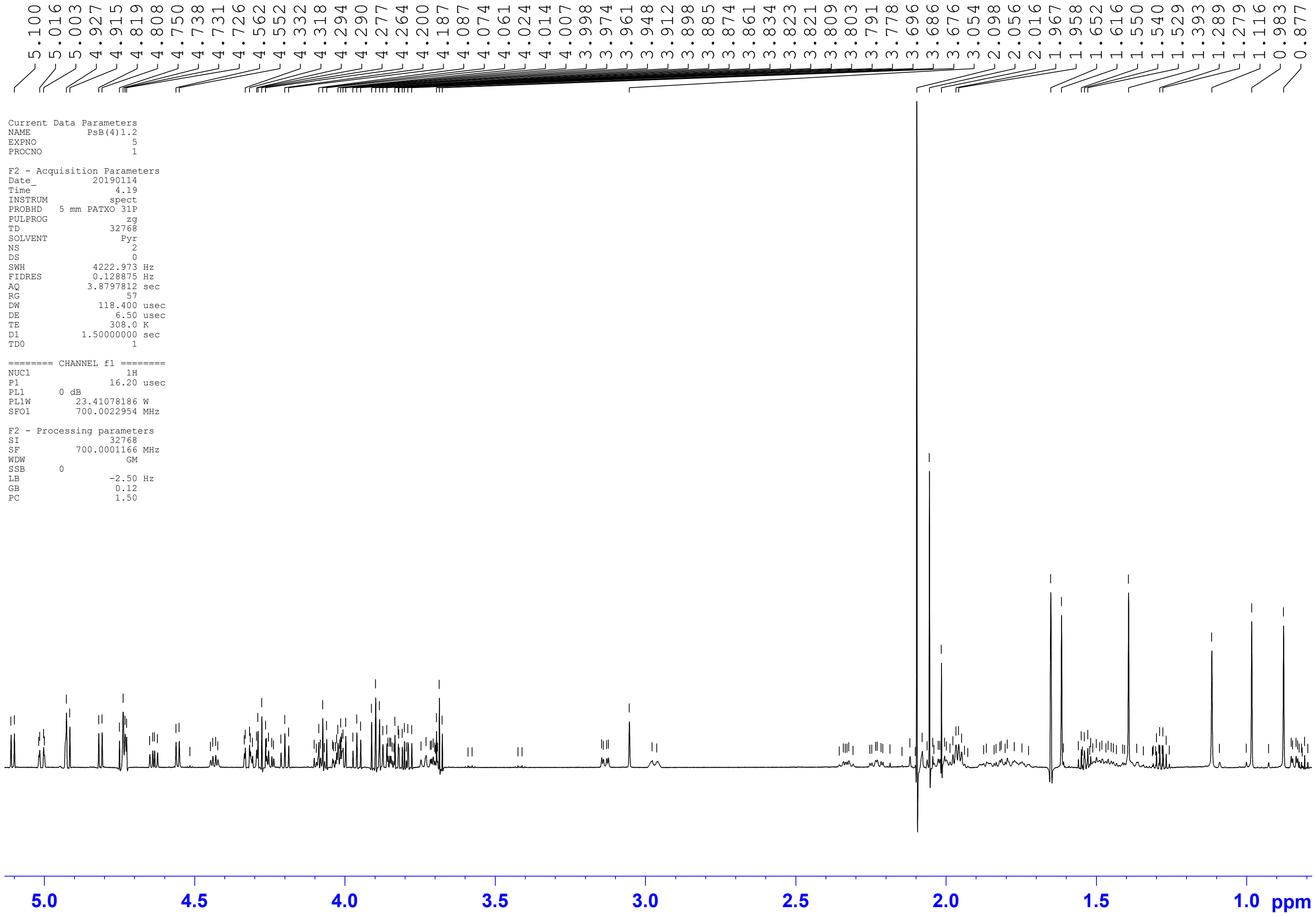

Fig. 2. The ^{1}H NMR (700.13 MHz) spectrum of psolusoside B (**1**) in C_5D_5N/D_2O (4/1)

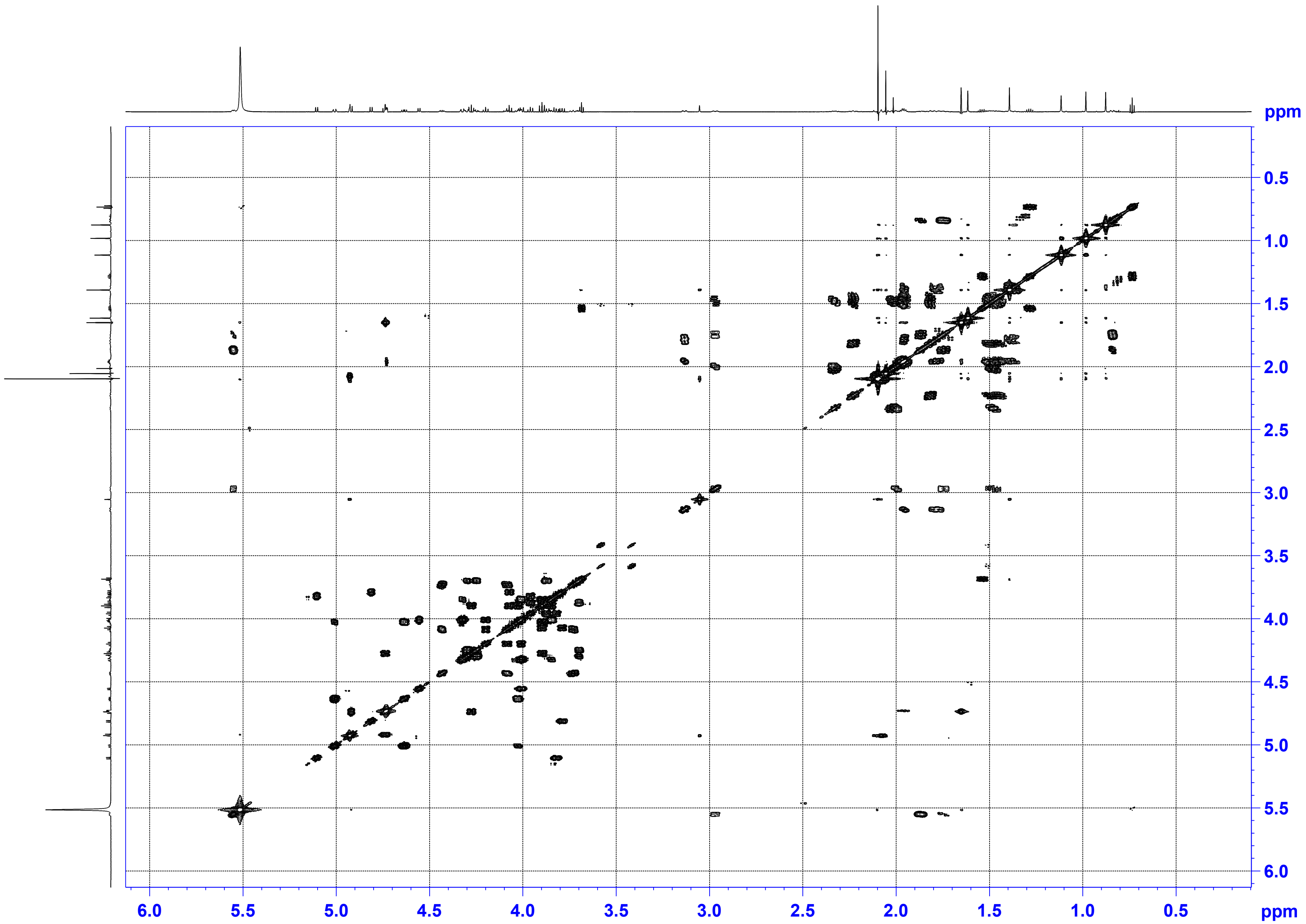

Fig. 3. The COSY (700.13 MHz) spectrum of psolusoside B (**1**) in C_5D_5N/D_2O (4/1)

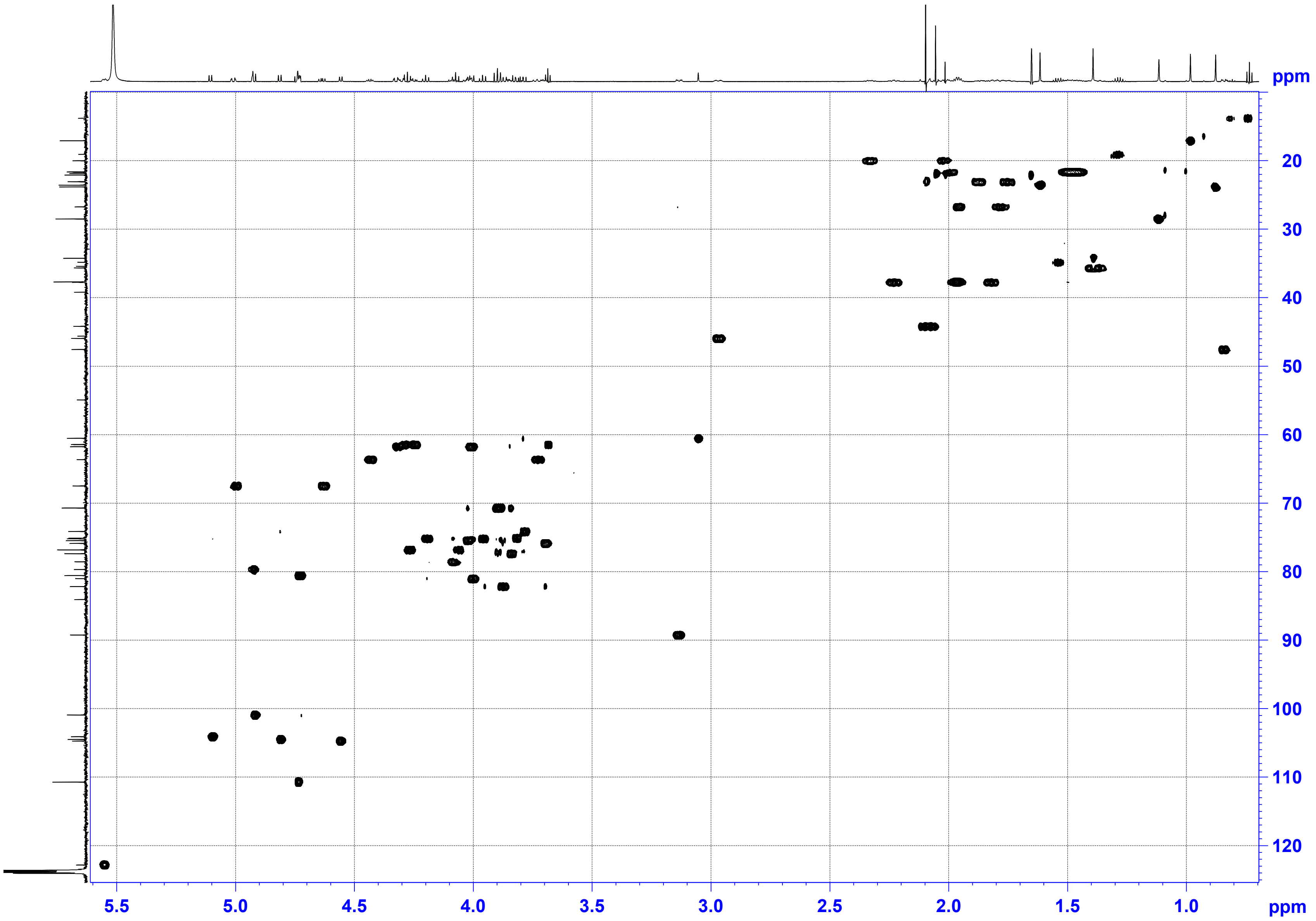

Fig. 4. The HSQC (700.13 MHz) spectrum of psolusoside B (**1**) in C_5D_5N/D_2O (4/1)

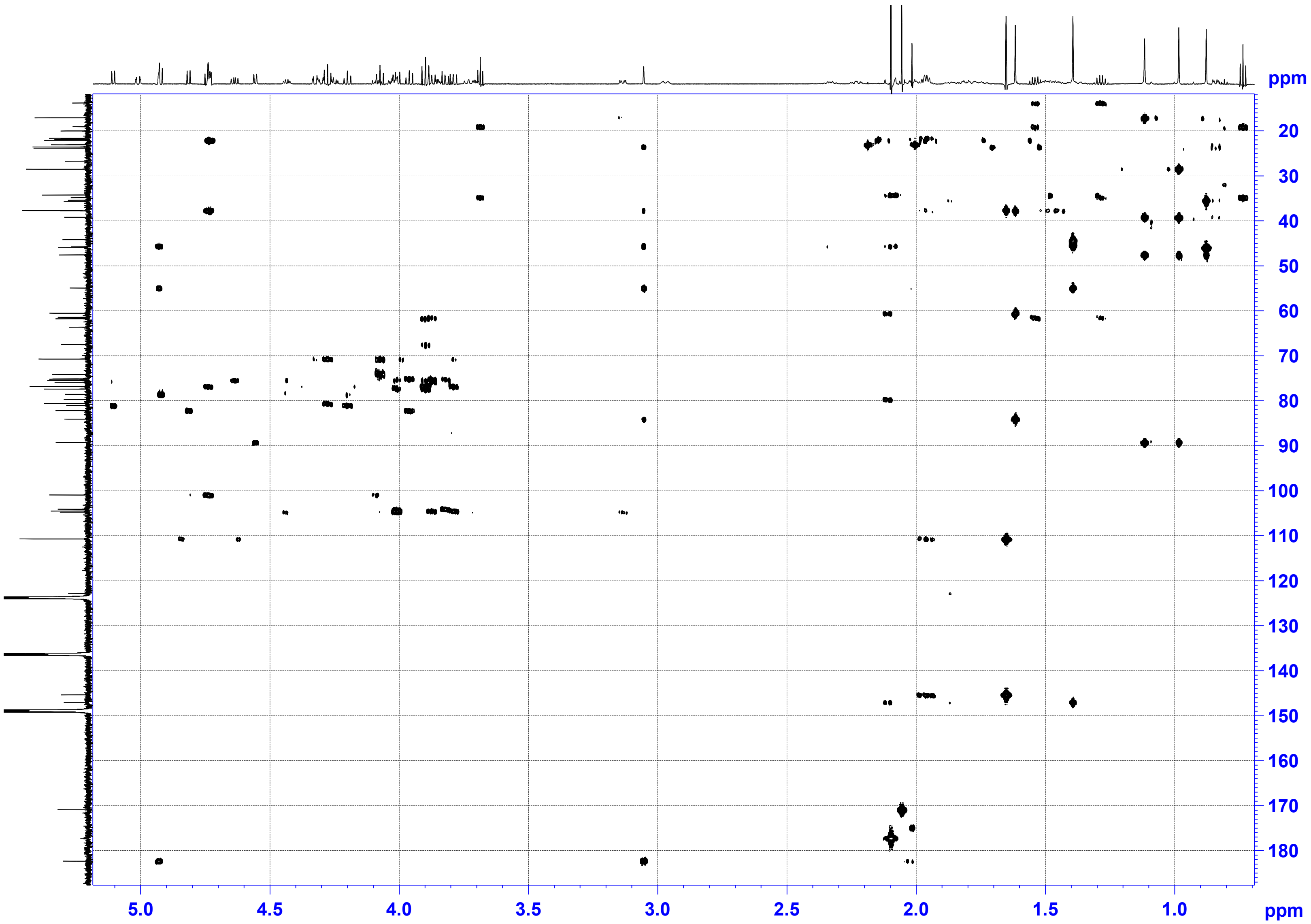

Fig. 5. The HMBC (700.13 MHz) spectrum of psolusoside B (**1**) in C_5D_5N/D_2O (4/1)

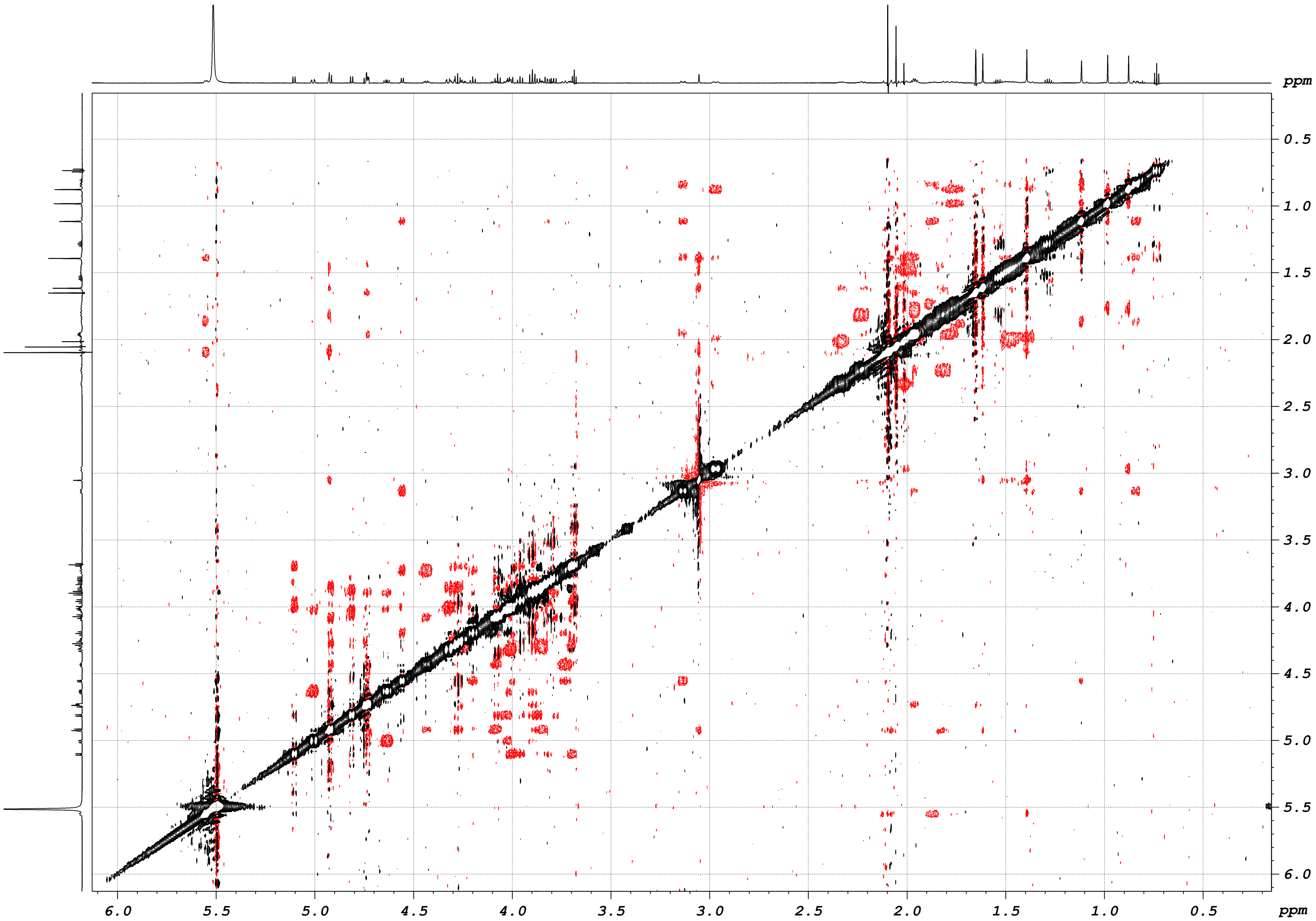

Fig. 6. The ROESY (500.13 MHz) spectrum of psolusoside B (**1**) in C_5D_5N/D_2O (4/1)

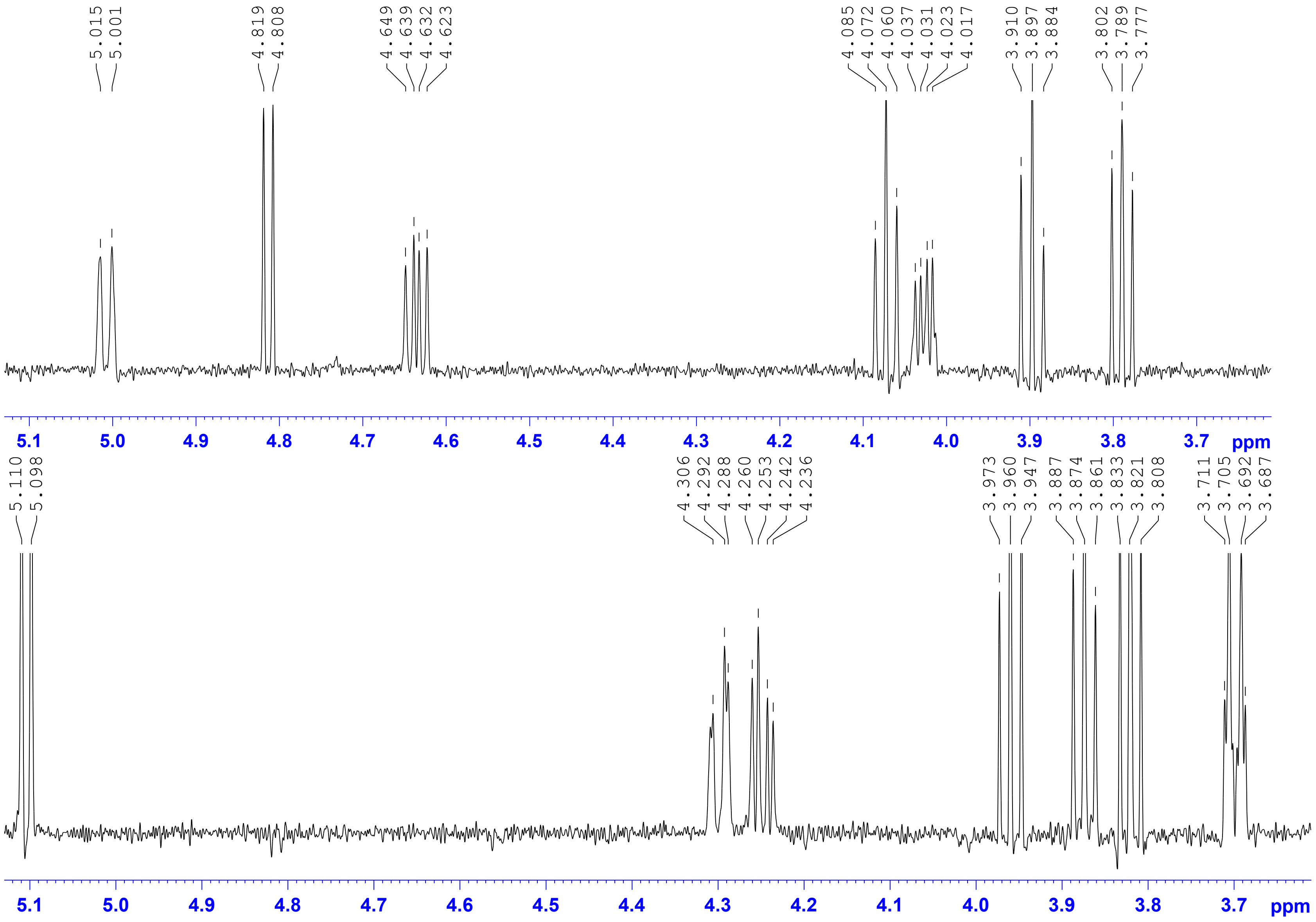

Fig. 7. 1 D TOCSY (700.13 MHz) spectra of psolusoside B (**1**) in C_5D_5N/D_2O (4/1)

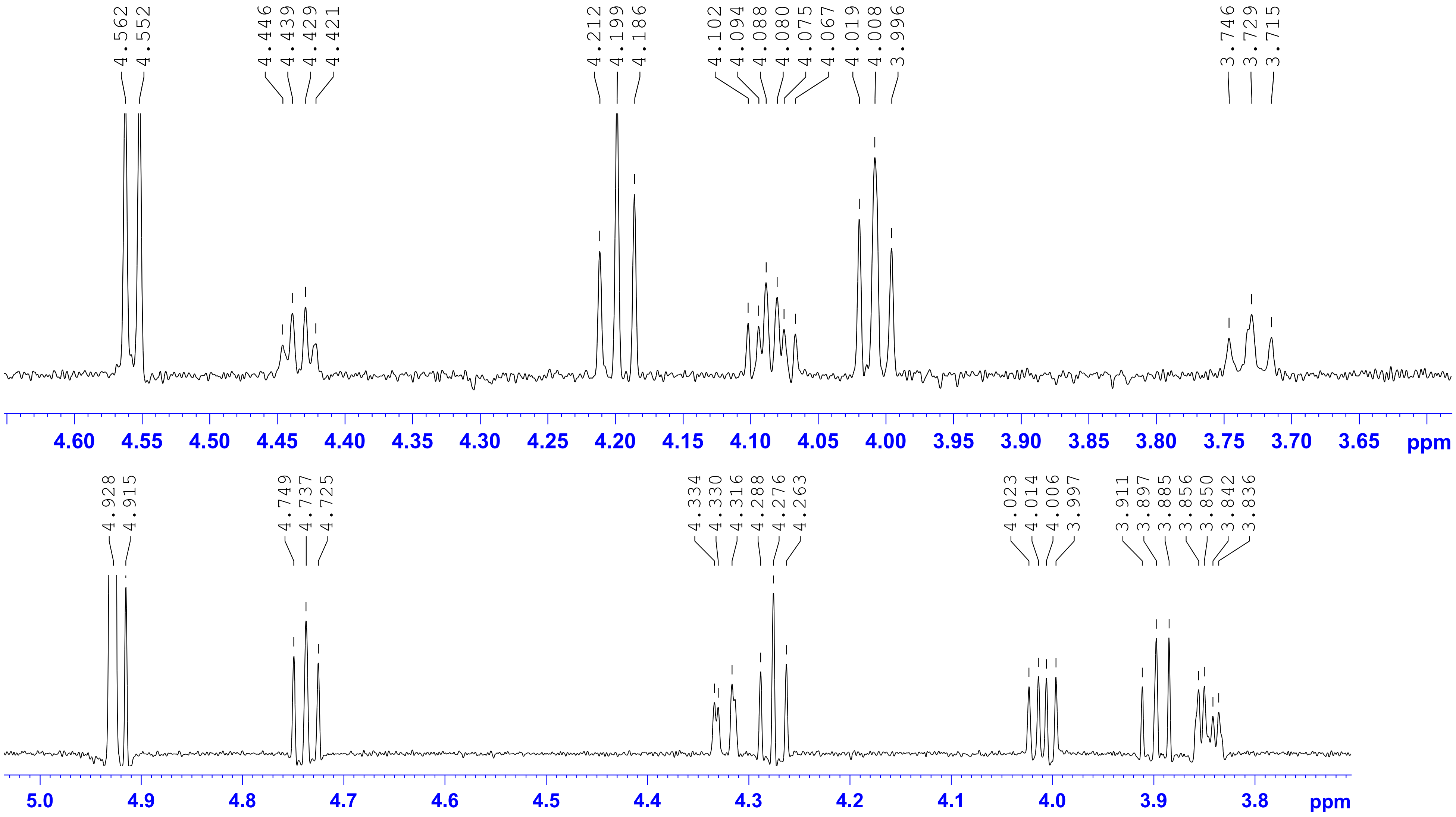

Fig. 8. 1 D TOCSY (700.13 MHz) spectra of psolusoside B (**1**) in C_5D_5N/D_2O (4/1)

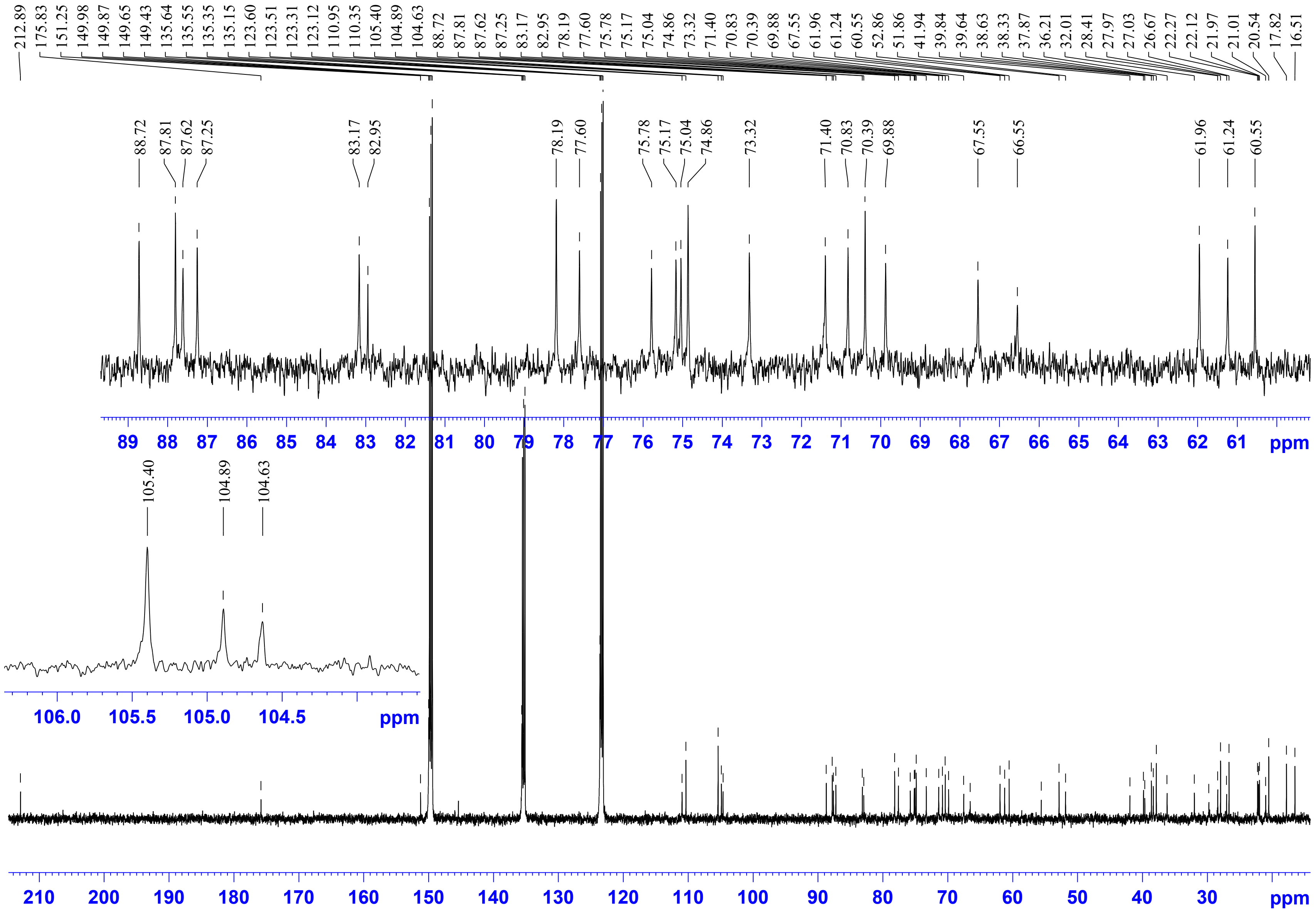

Fig. 9. The ^{13}C NMR (176.04 MHz) spectrum of psolusoside E (**2**) in in C_5D_5N/D_2O (4/1)

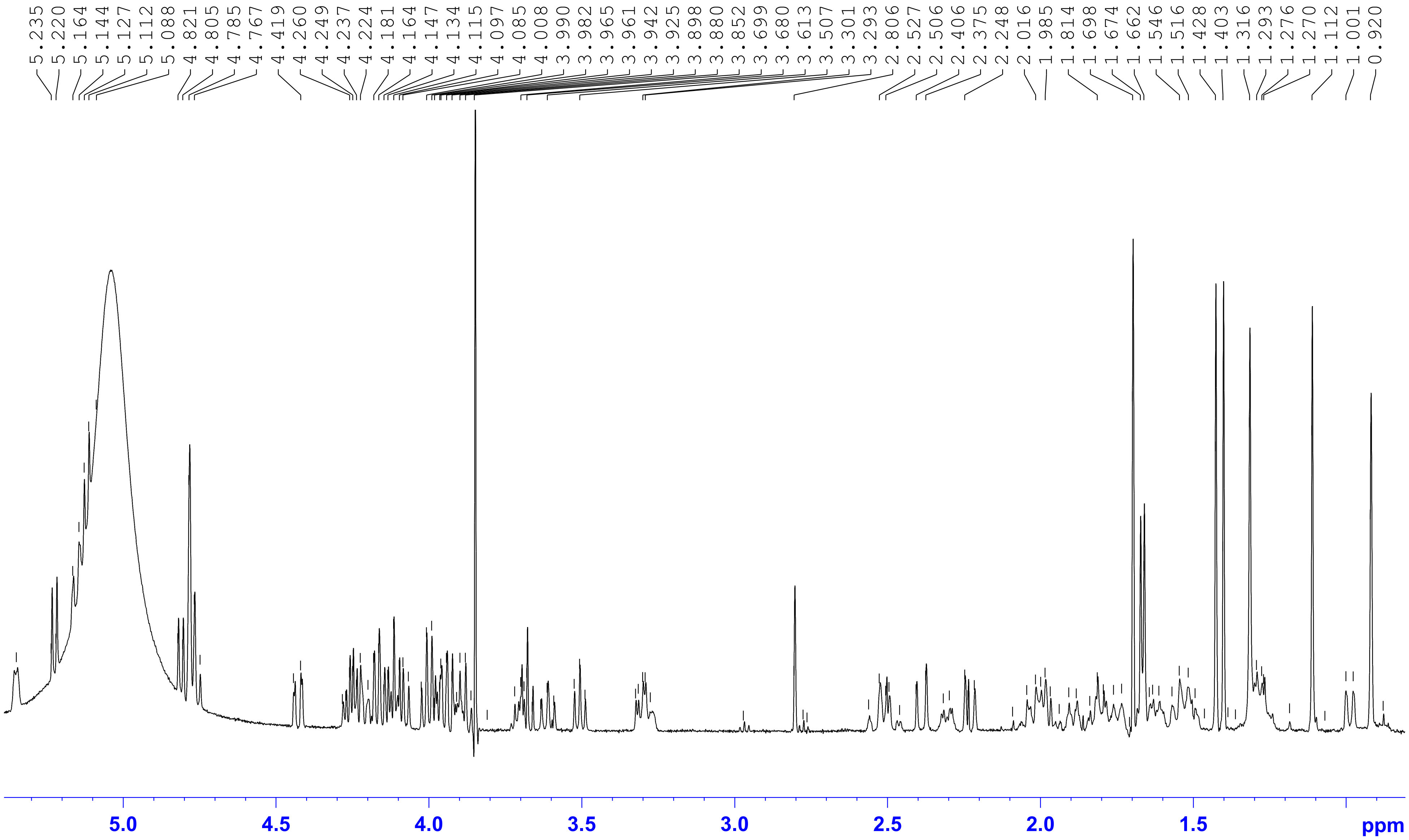

Fig. 10. The ^{1}H NMR (700.13 MHz) spectrum of psolusoside E (**2**) in C_5D_5N/D_2O (4/1)

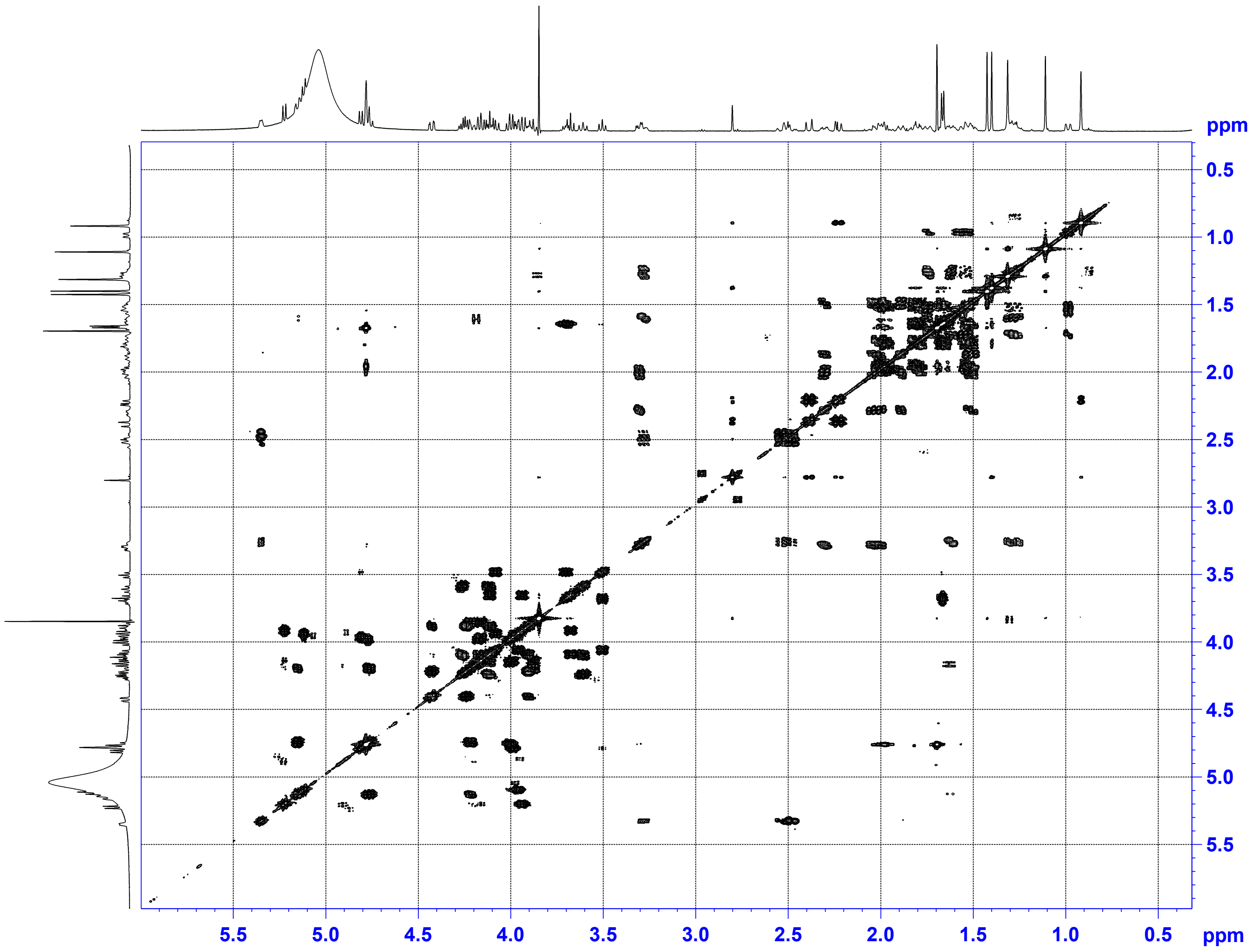

Fig. 11. The COSY (700.13 MHz) spectrum of psolusoside E (**2**) in C_5D_5N/D_2O (4/1)

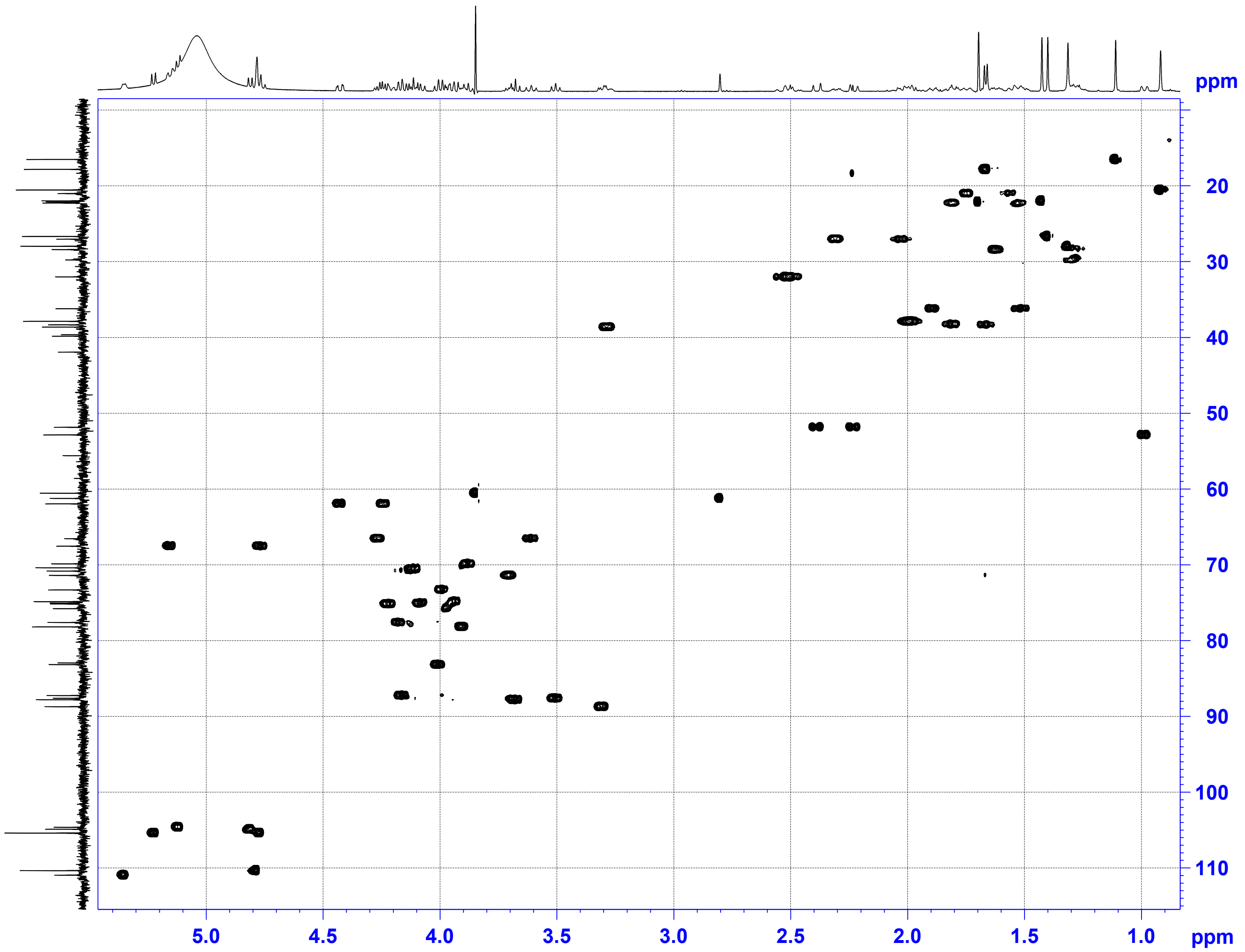

Fig. 12. The HSQC (700.13 MHz) spectrum of psolusoside E (**2**) in C_5D_5N/D_2O (4/1)

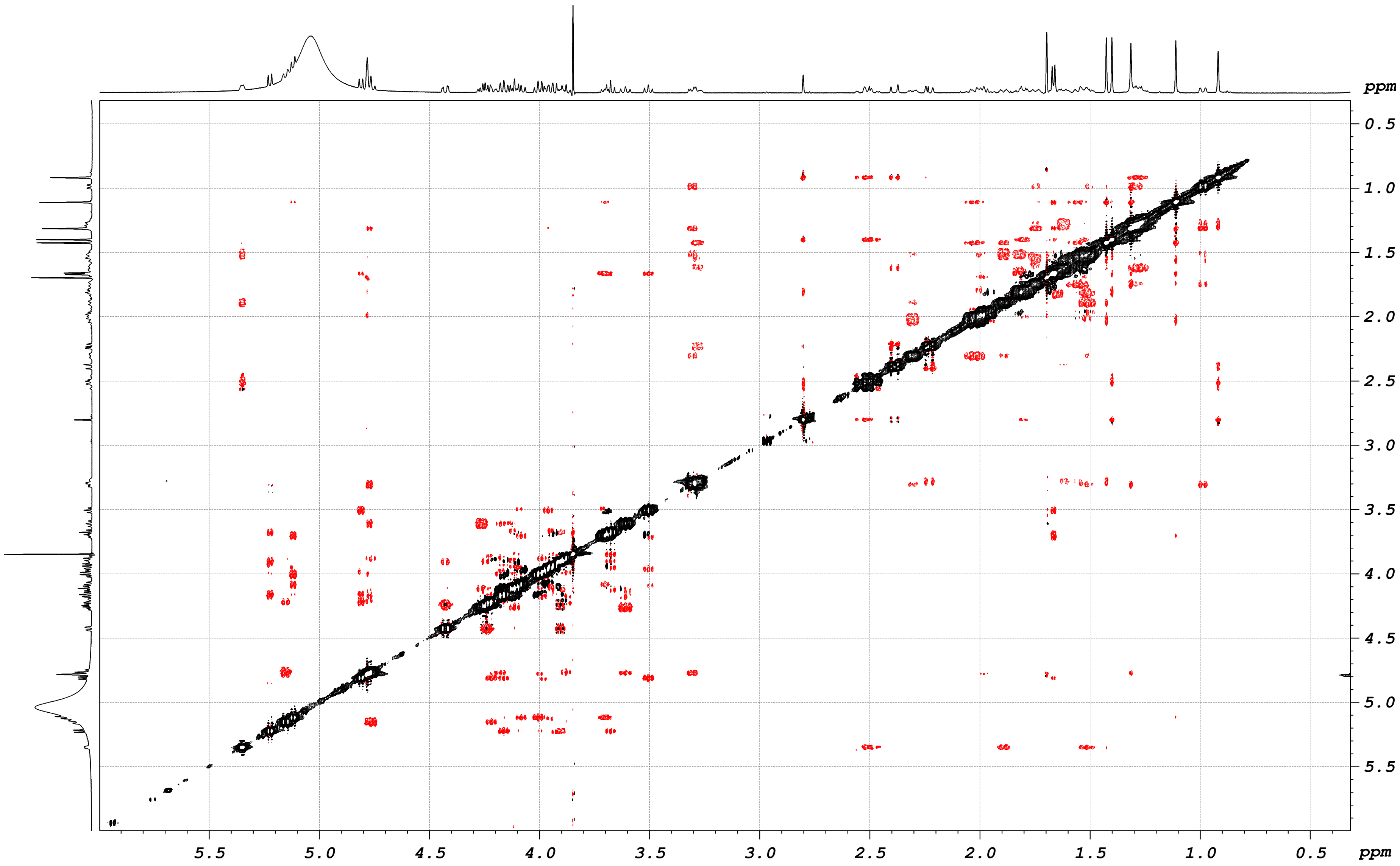

Fig. 13. The ROESY (500.13 MHz) spectrum of psolusoside E (**2**) in C_5D_5N/D_2O (4/1)

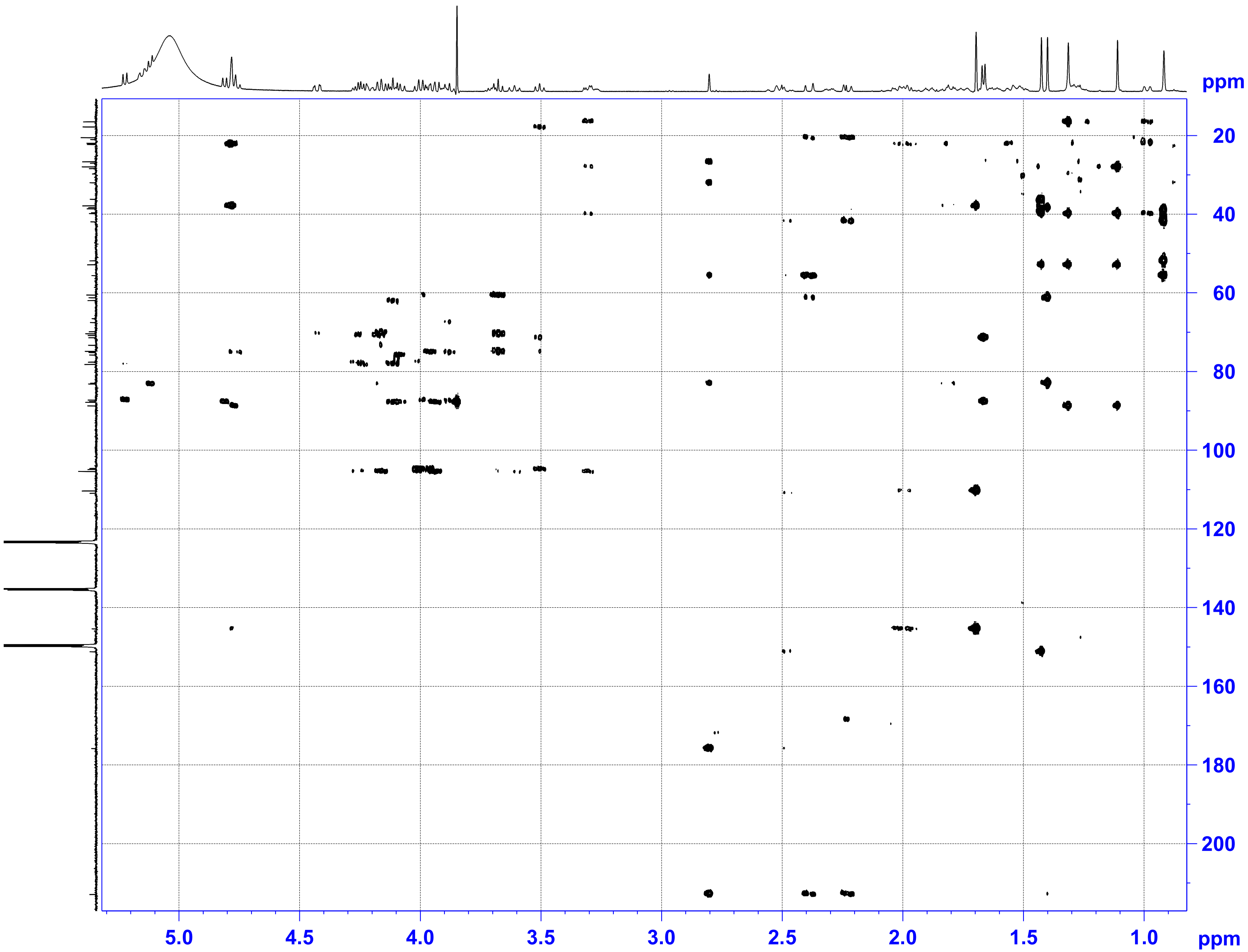

Fig. 14. The HMBC (700.13 MHz) spectrum of psolusoside E (**2**) in C_5D_5N/D_2O (4/1)

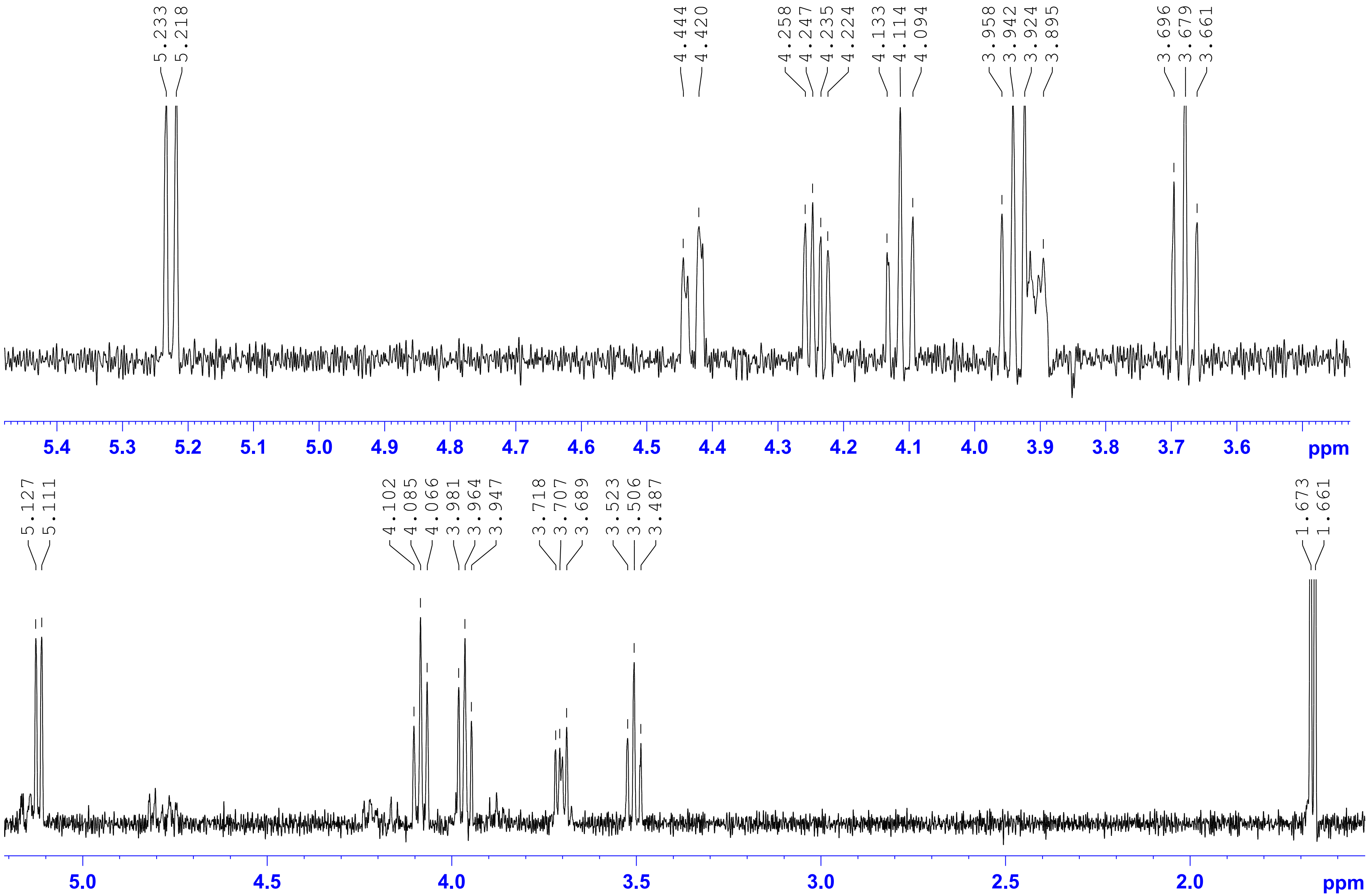

Fig. 15. 1D TOCSY (700.13 MHz) spectra of psolusoside E (**2**) in C_5D_5N/D_2O (4/1)

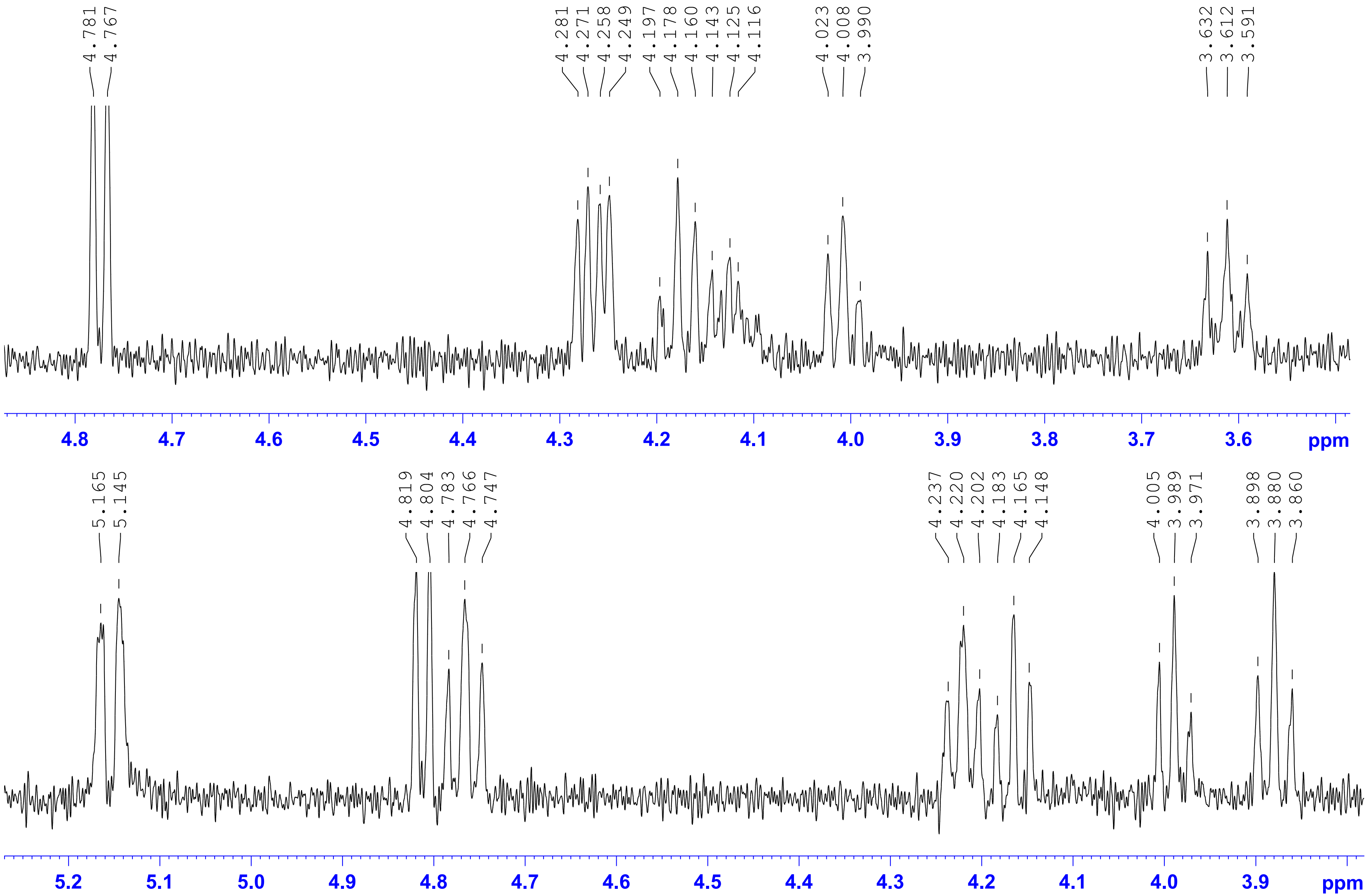

Fig. 16. 1D TOCSY (700.13 MHz) spectra of psolusoside E (**2**) in C_5D_5N/D_2O (4/1)

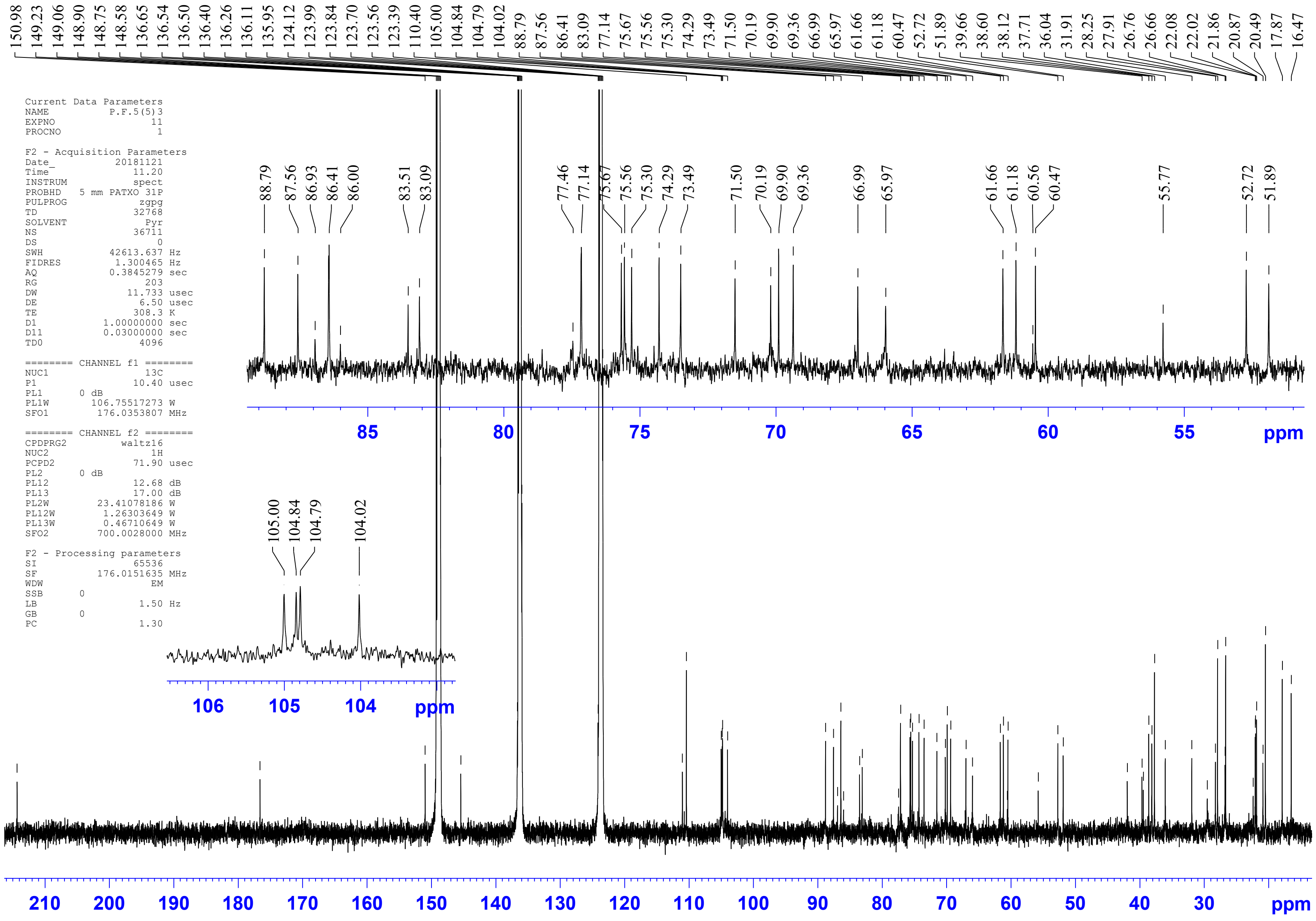

Fig. 17. The ^{13}C NMR (176.04 MHz) spectrum of psolusoside F (**3**) in in C_5D_5N/D_2O (4/1)

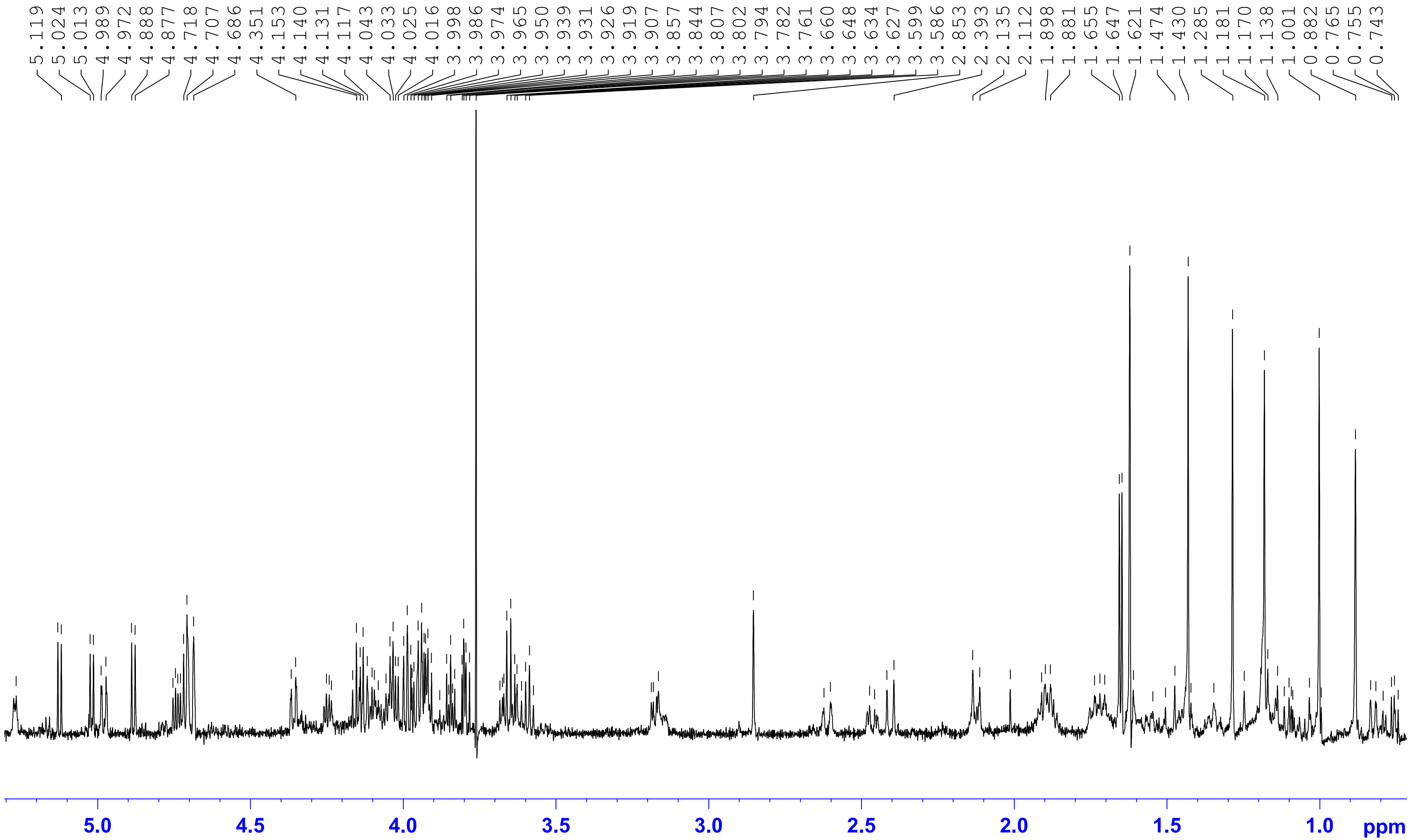

Fig. 18. The ^{1}H NMR (700.13 MHz) spectrum of psolusoside F (**3**) in C_5D_5N/D_2O (4/1)

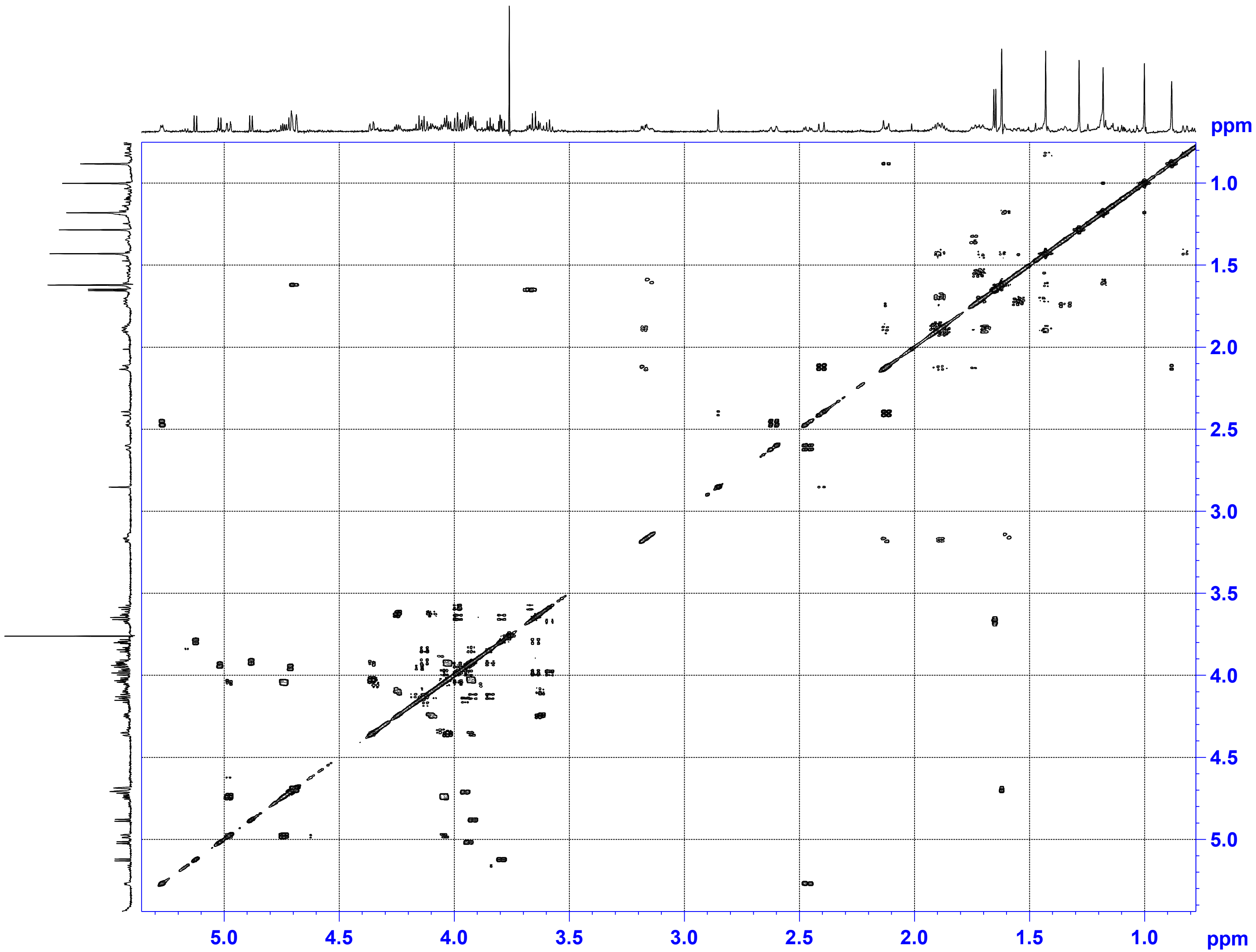

Fig. 19. The COSY (700.13 MHz) spectrum of psolusoside F (**3**) in C_5D_5N/D_2O (4/1)

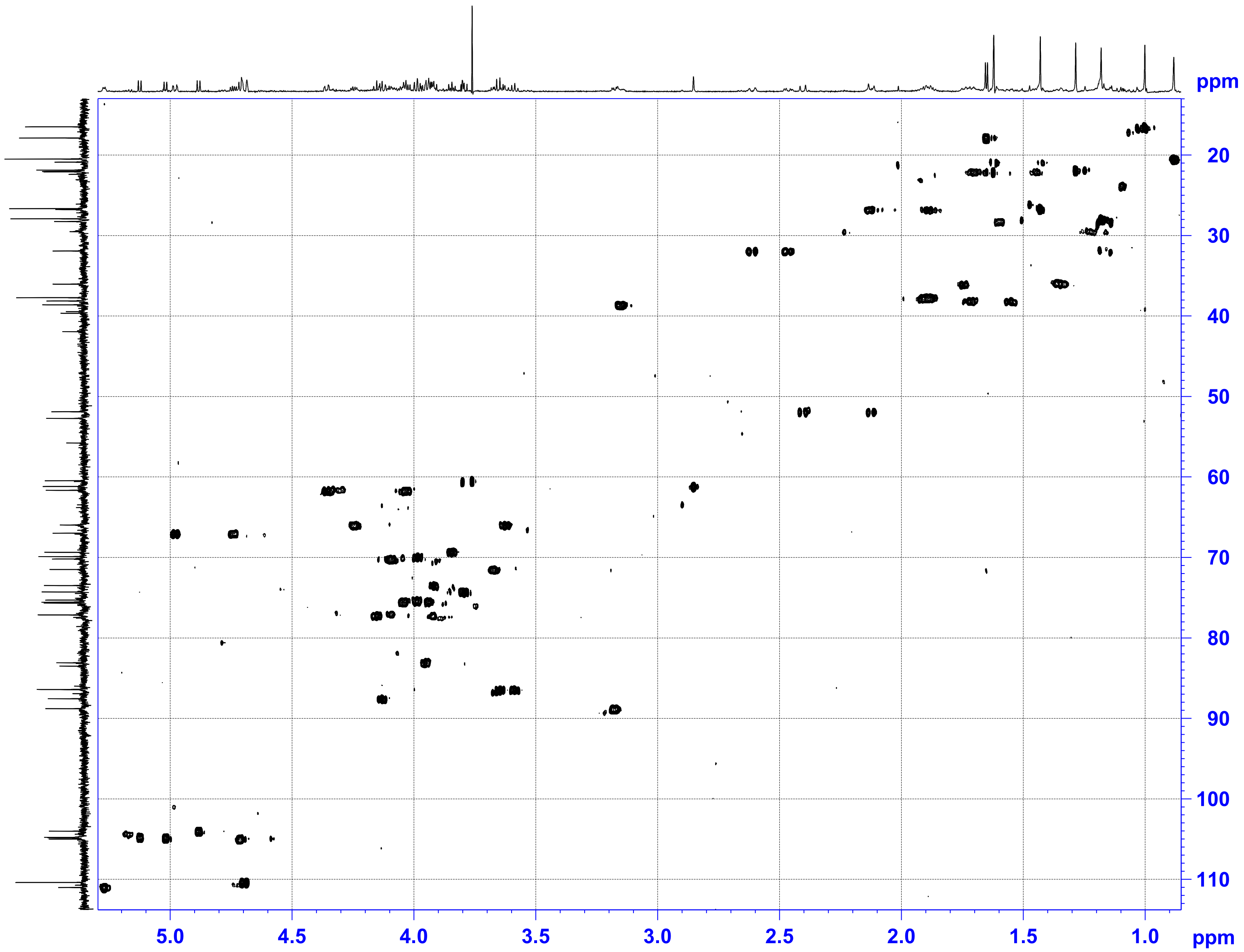

Fig. 20. The HSQC (700.13 MHz) spectrum of psolusoside F (**3**) in C_5D_5N/D_2O (4/1)

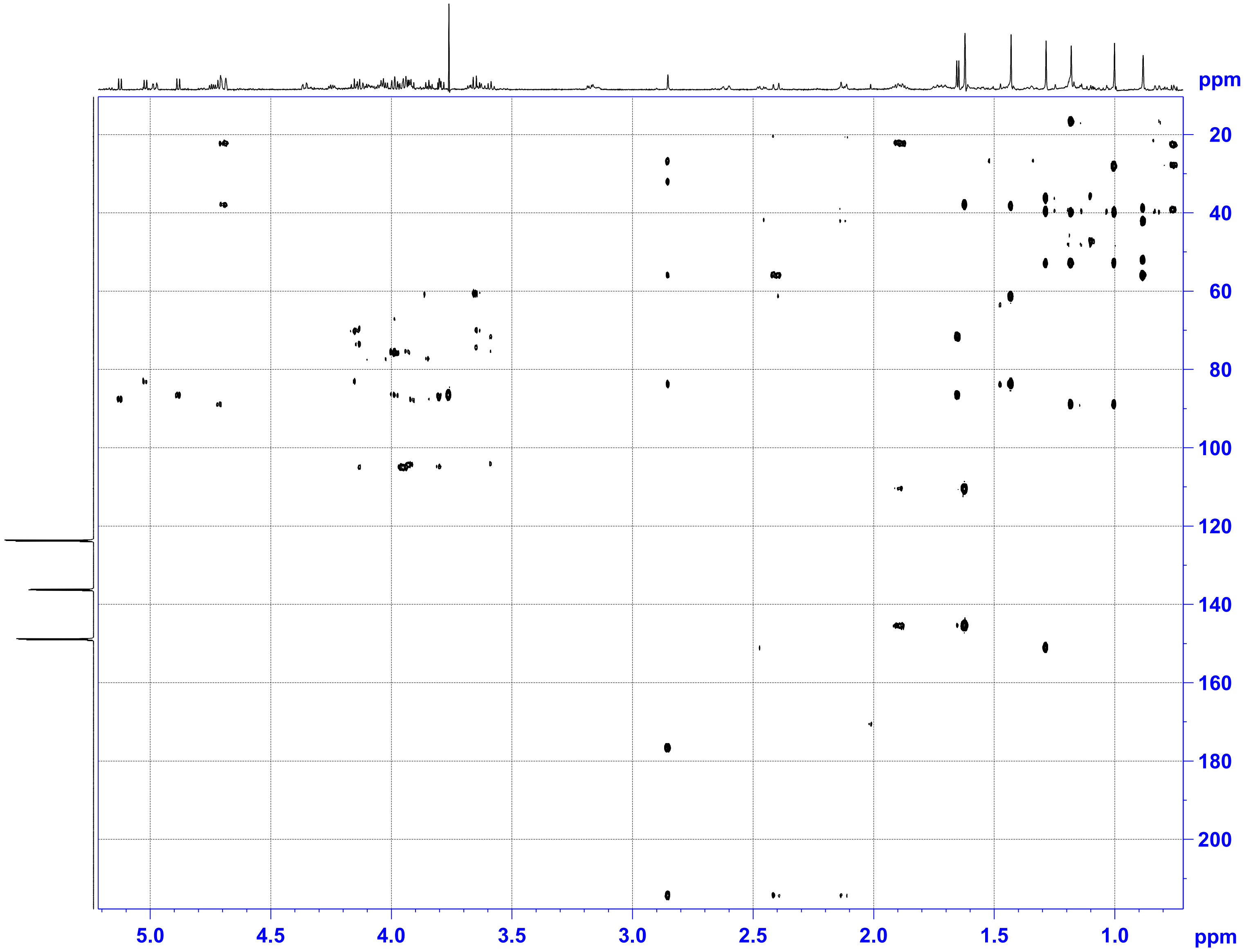

Fig. 21. The HMBC (700.13 MHz) spectrum of psolusoside F (**3**) in C_5D_5N/D_2O (4/1)

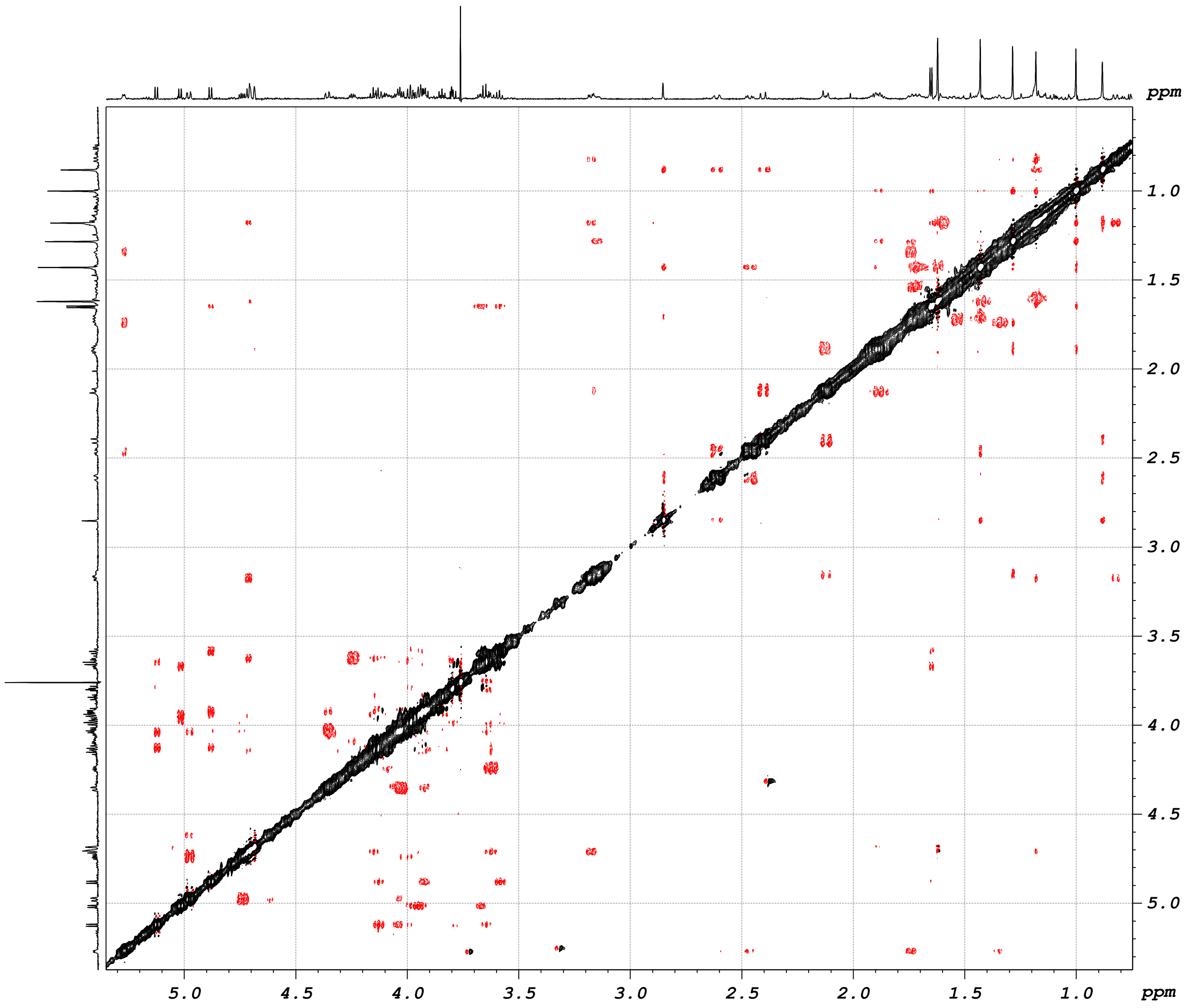

Fig. 22. The ROESY (500.13 MHz) spectrum of psolusoside F (**3**) in C_5D_5N/D_2O (4/1)

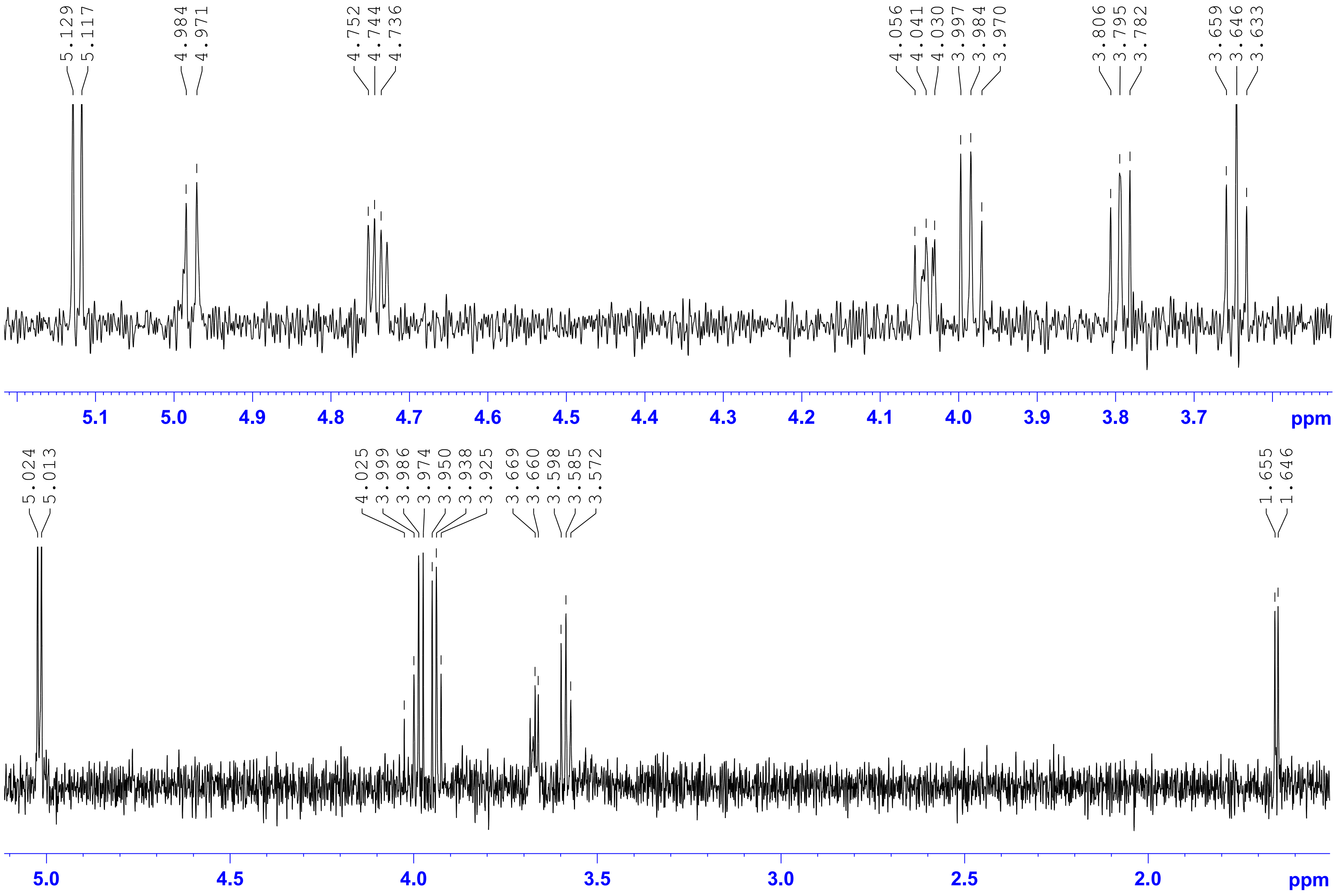

Fig. 23. 1D TOCSY (700.13 MHz) spectra of psolusoside F (**3**) in C_5D_5N/D_2O (4/1)

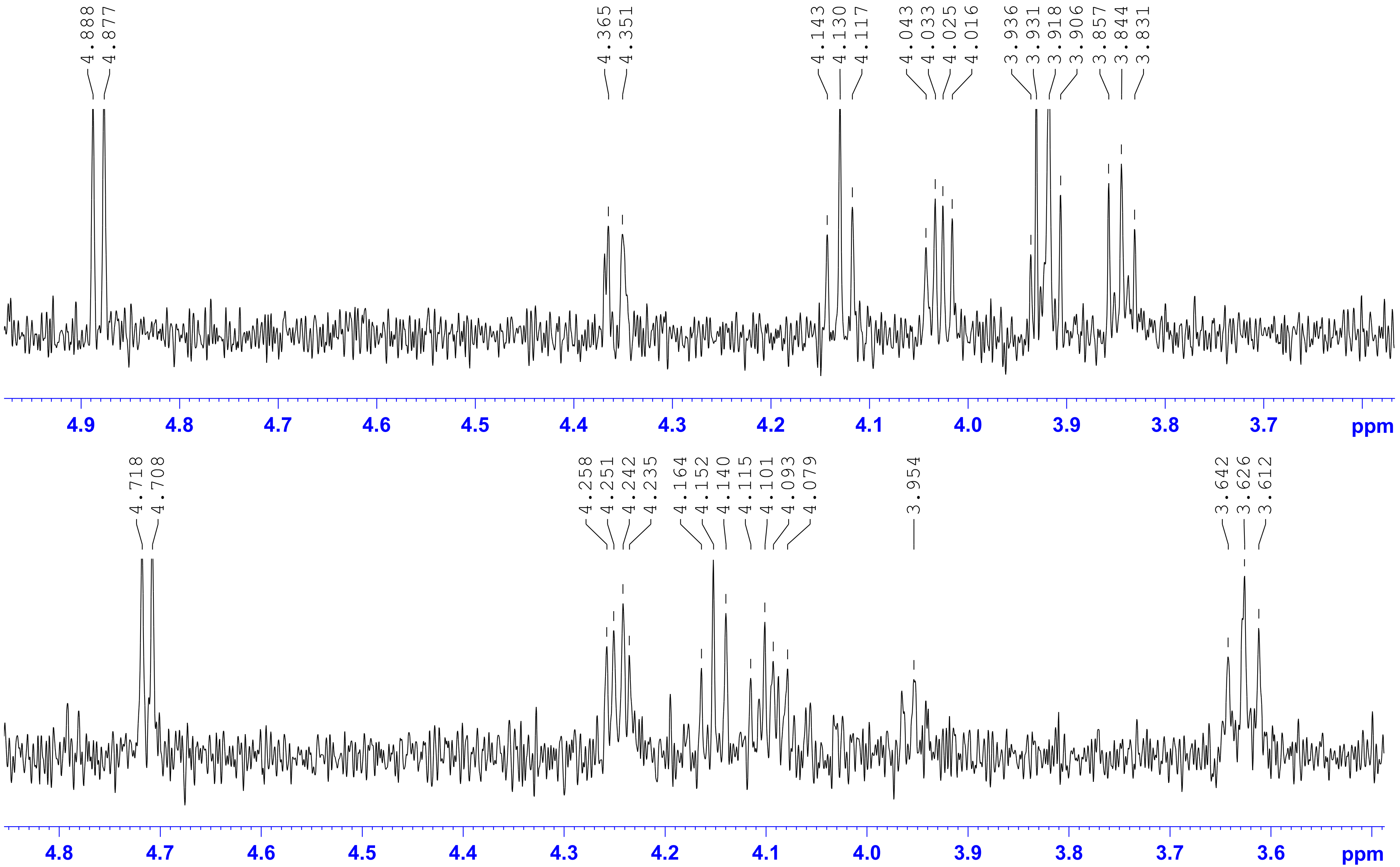

Fig. 24. 1D TOCSY (700.13 MHz) spectra of psolusoside F (**3**) in C_5D_5N/D_2O (4/1)

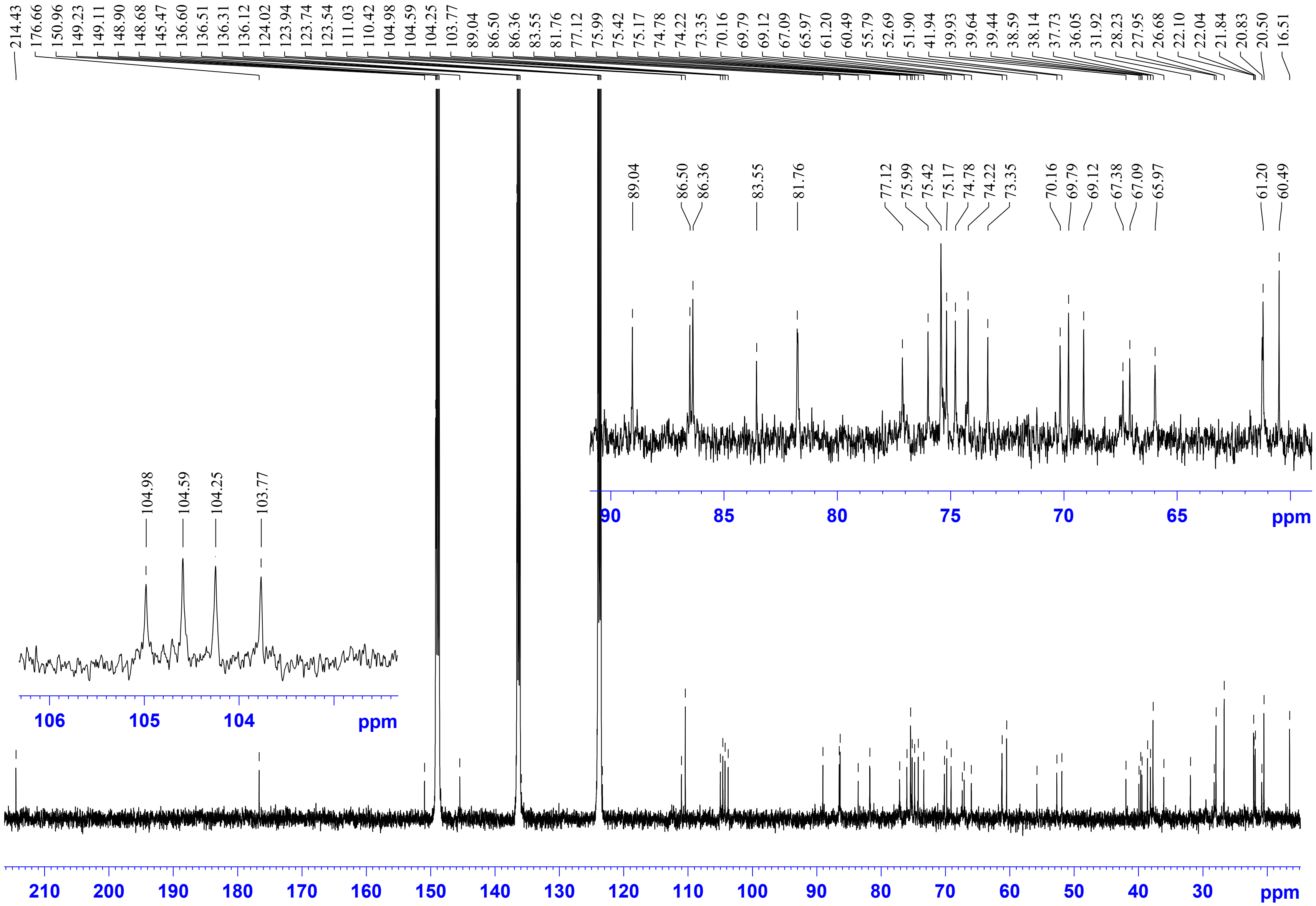

Fig. 25. The ^{13}C NMR (176.04 MHz) spectrum of psolusoside G (**4**) in in C_5D_5N/D_2O (4/1)

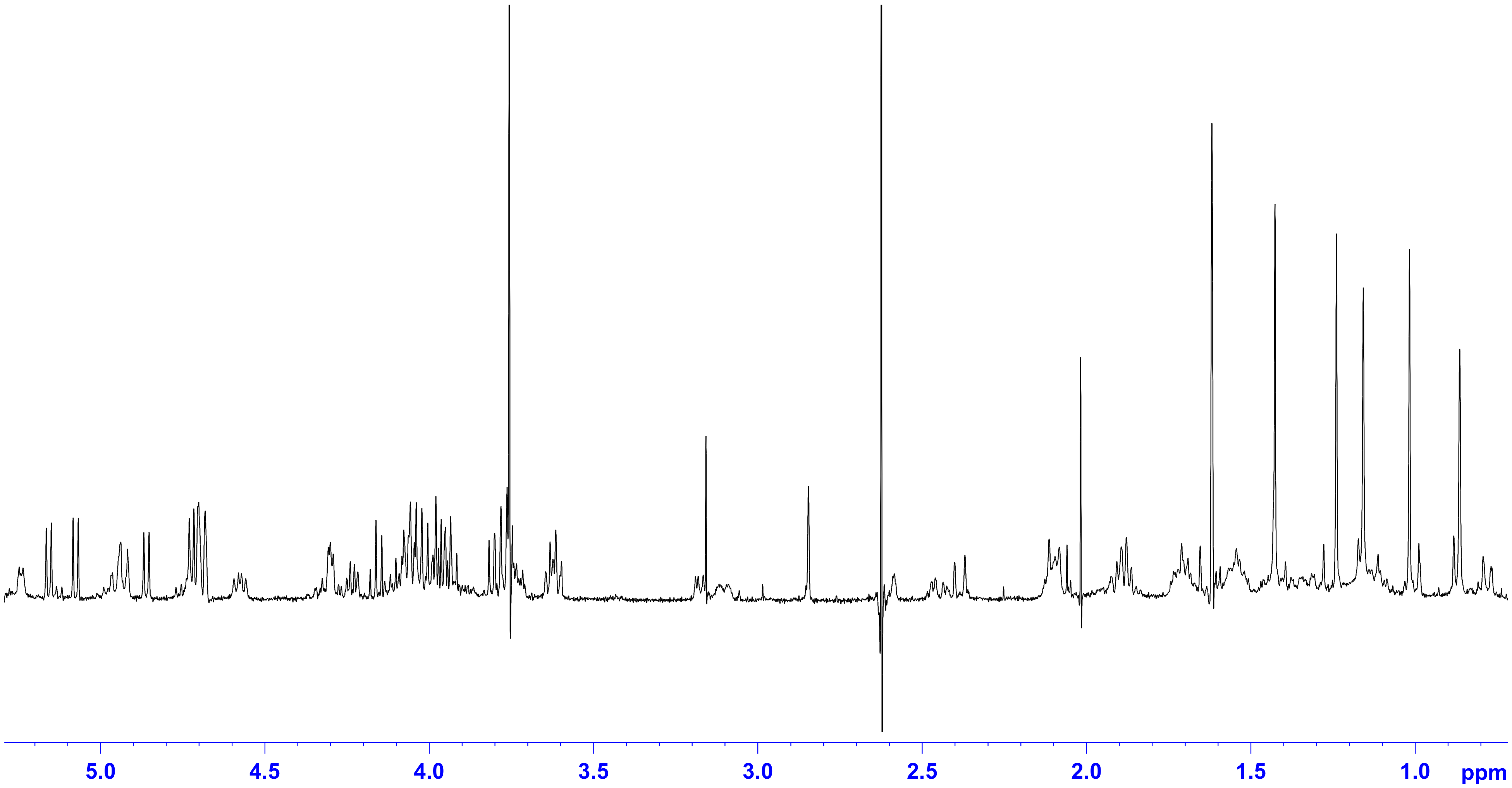

Fig. 26. The ^{1}H NMR (700.13 MHz) spectrum of psolusoside G (**4**) in C_5D_5N/D_2O (4/1)

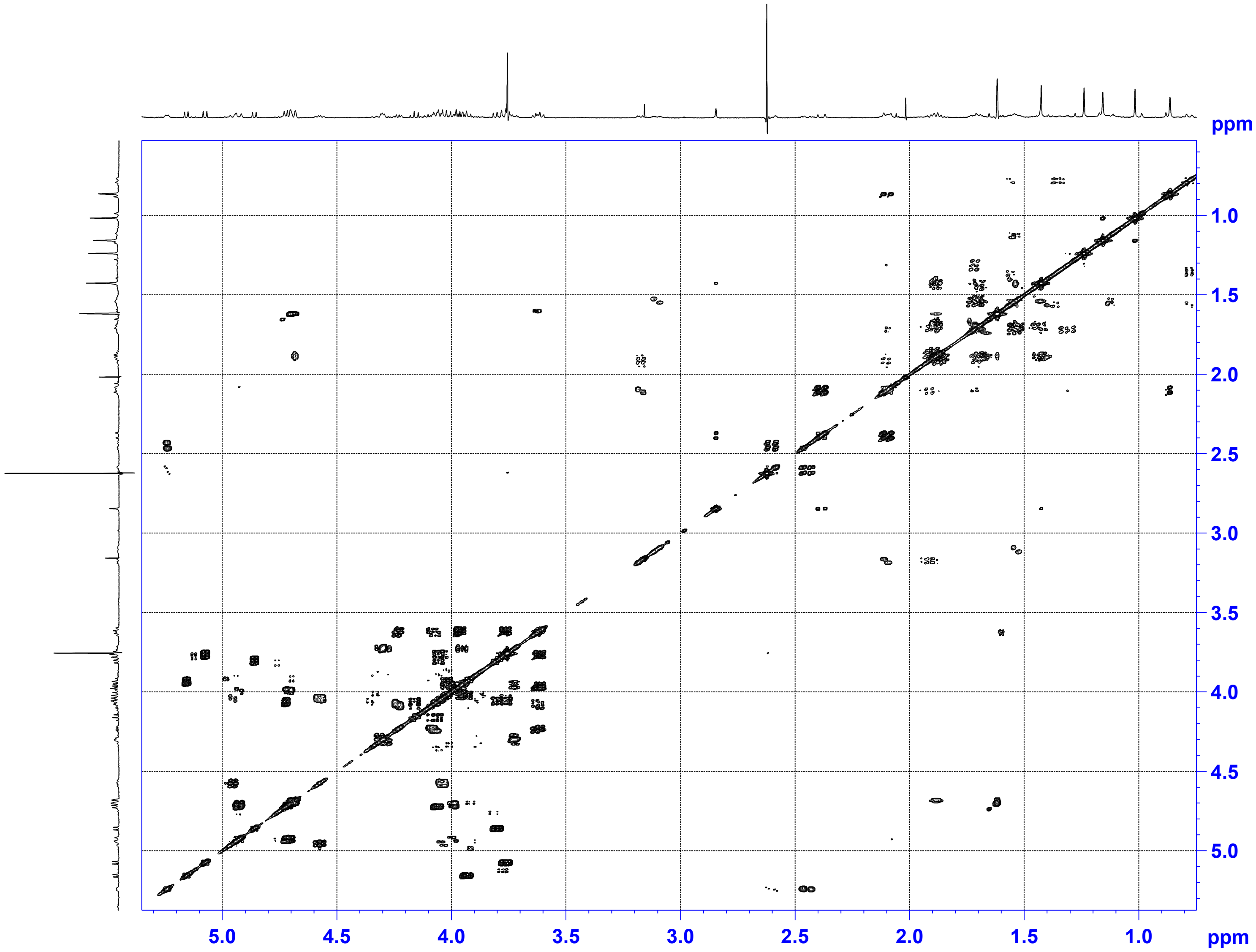

Fig. 27. The COSY (700.13 MHz) spectrum of psolusoside G (**4**) in C_5D_5N/D_2O (4/1)

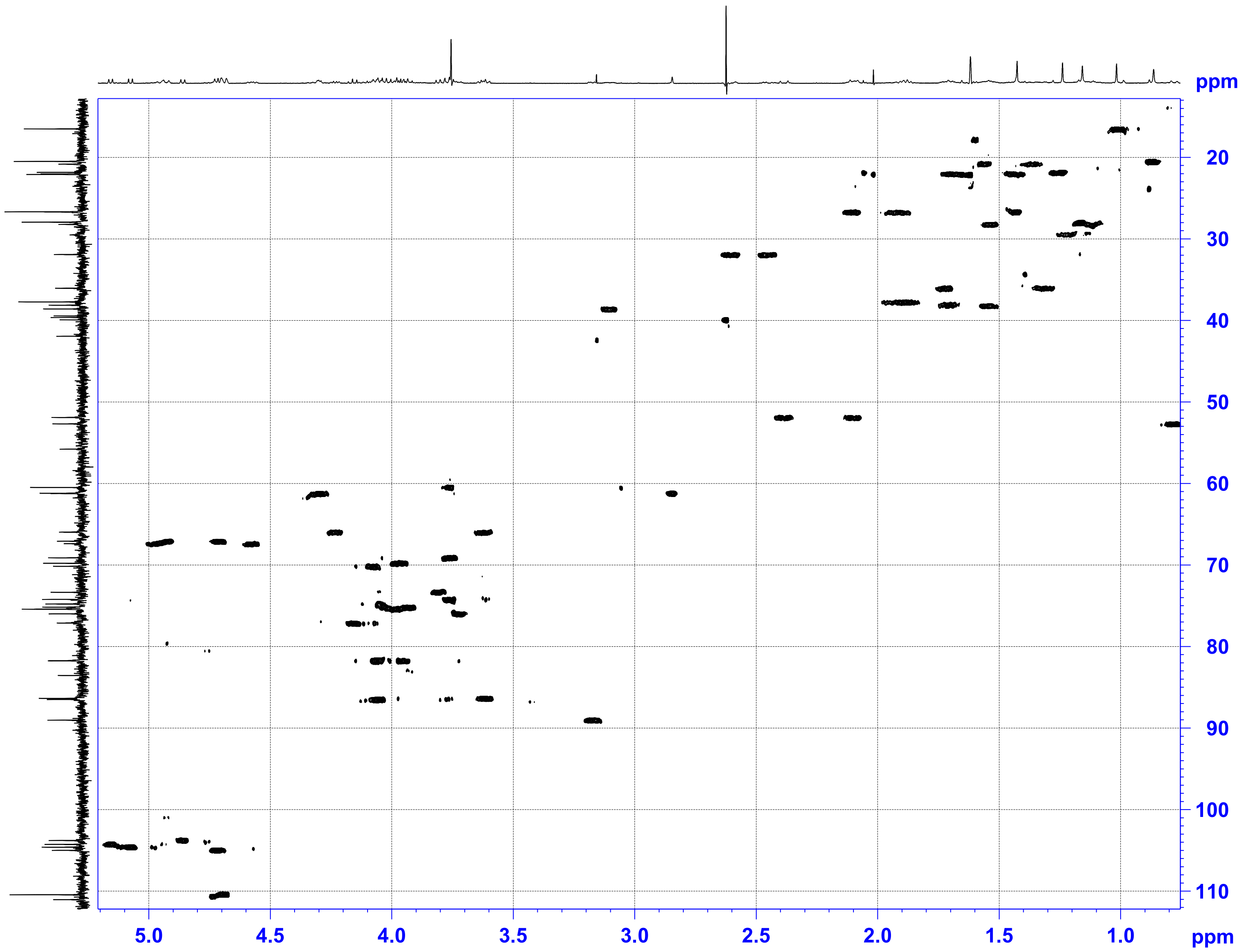

Fig. 28. The HSQC (700.13 MHz) spectrum of psolusoside G (**4**) in C_5D_5N/D_2O (4/1)

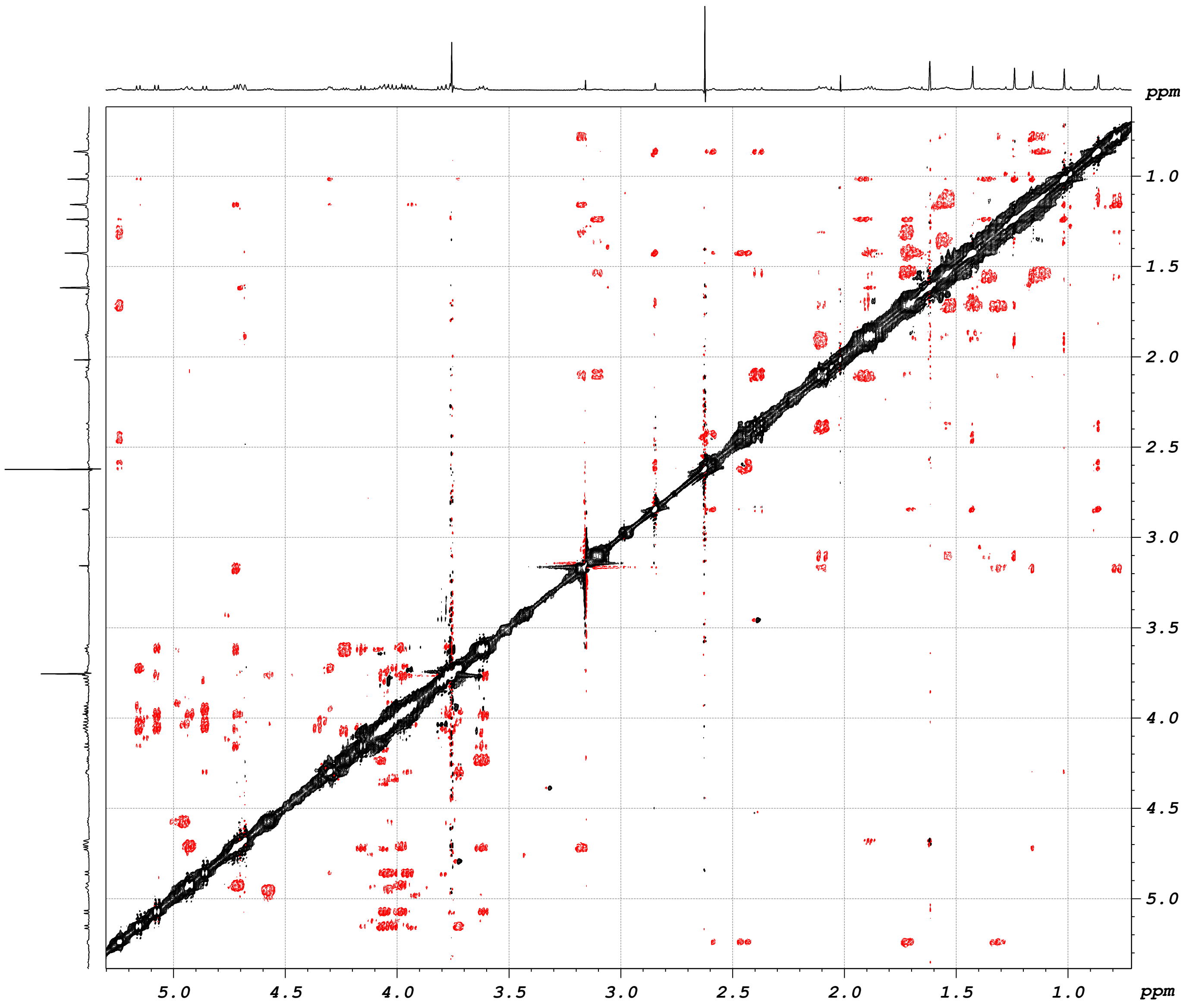

Fig. 29. The ROESY (500.13 MHz) spectrum of psolusoside G (**4**) in C_5D_5N/D_2O (4/1)

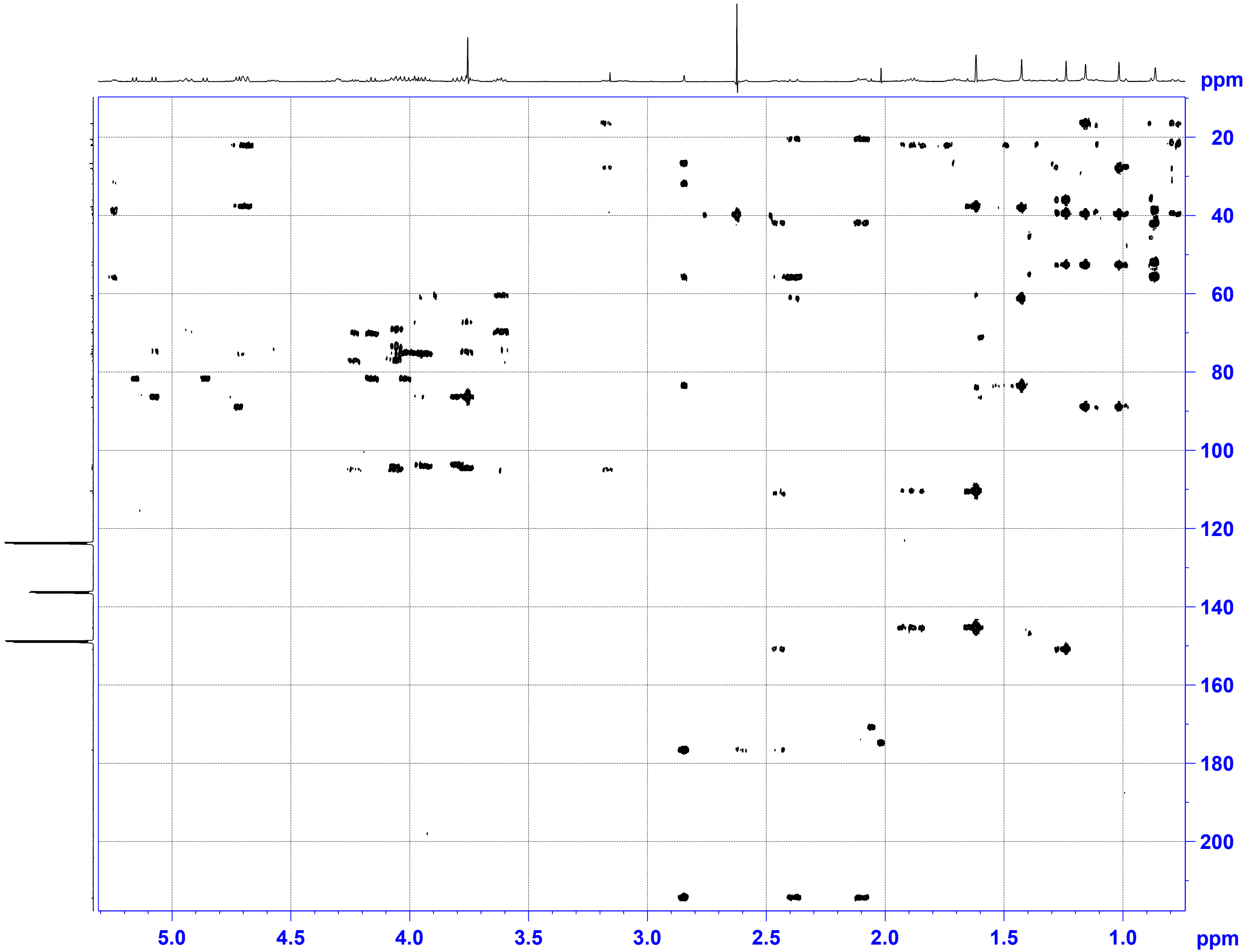

Fig. 30. The HMBC (700.13 MHz) spectrum of psolusoside G (**4**) in C_5D_5N/D_2O (4/1)

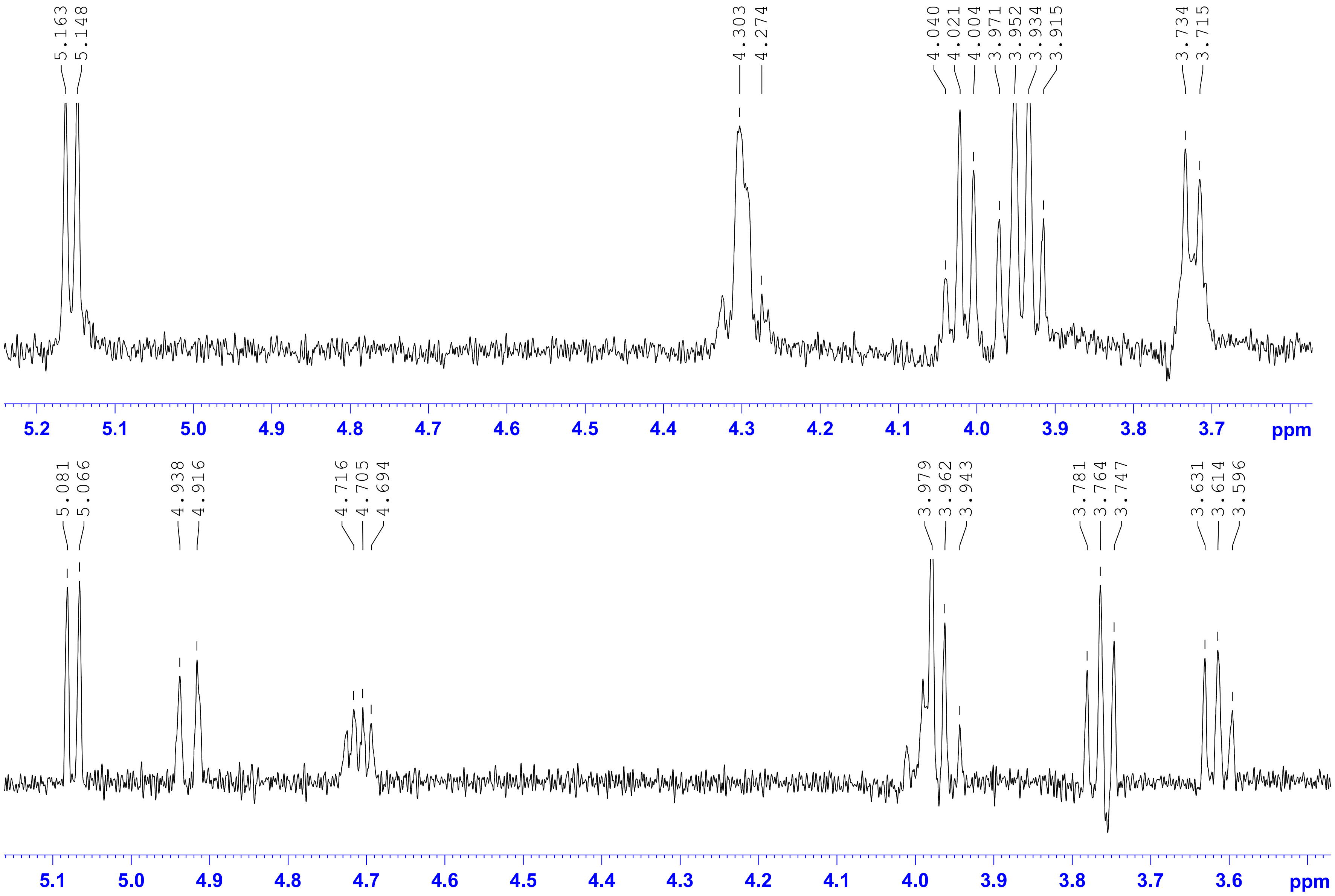

Fig. 31. 1D TOCSY (700.13 MHz) spectra of psolusoside G (**4**) in C_5D_5N/D_2O (4/1)

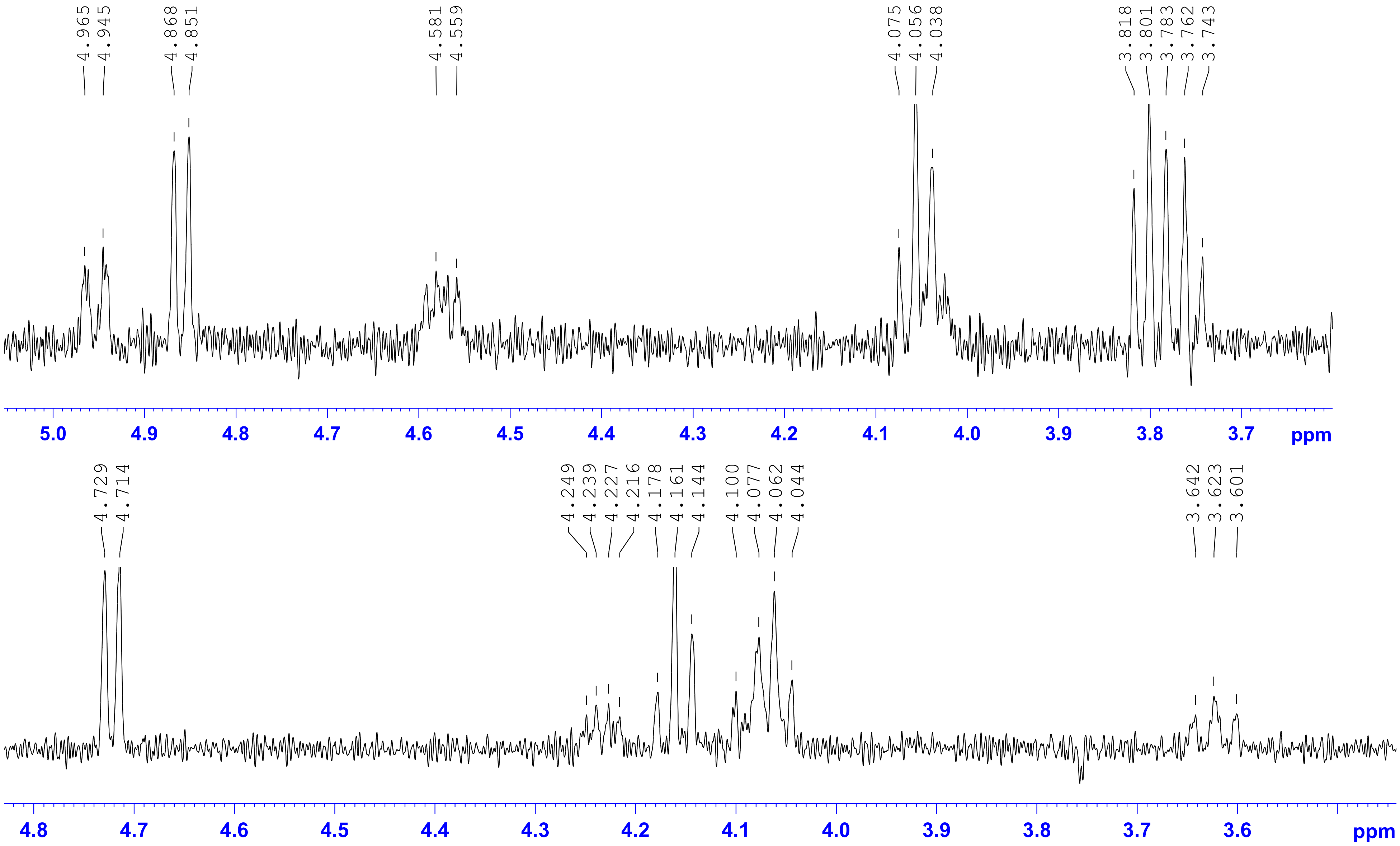

Fig. 32. 1D TOCSY (700.13 MHz) spectra of psolusoside G (**4**) in C_5D_5N/D_2O (4/1)

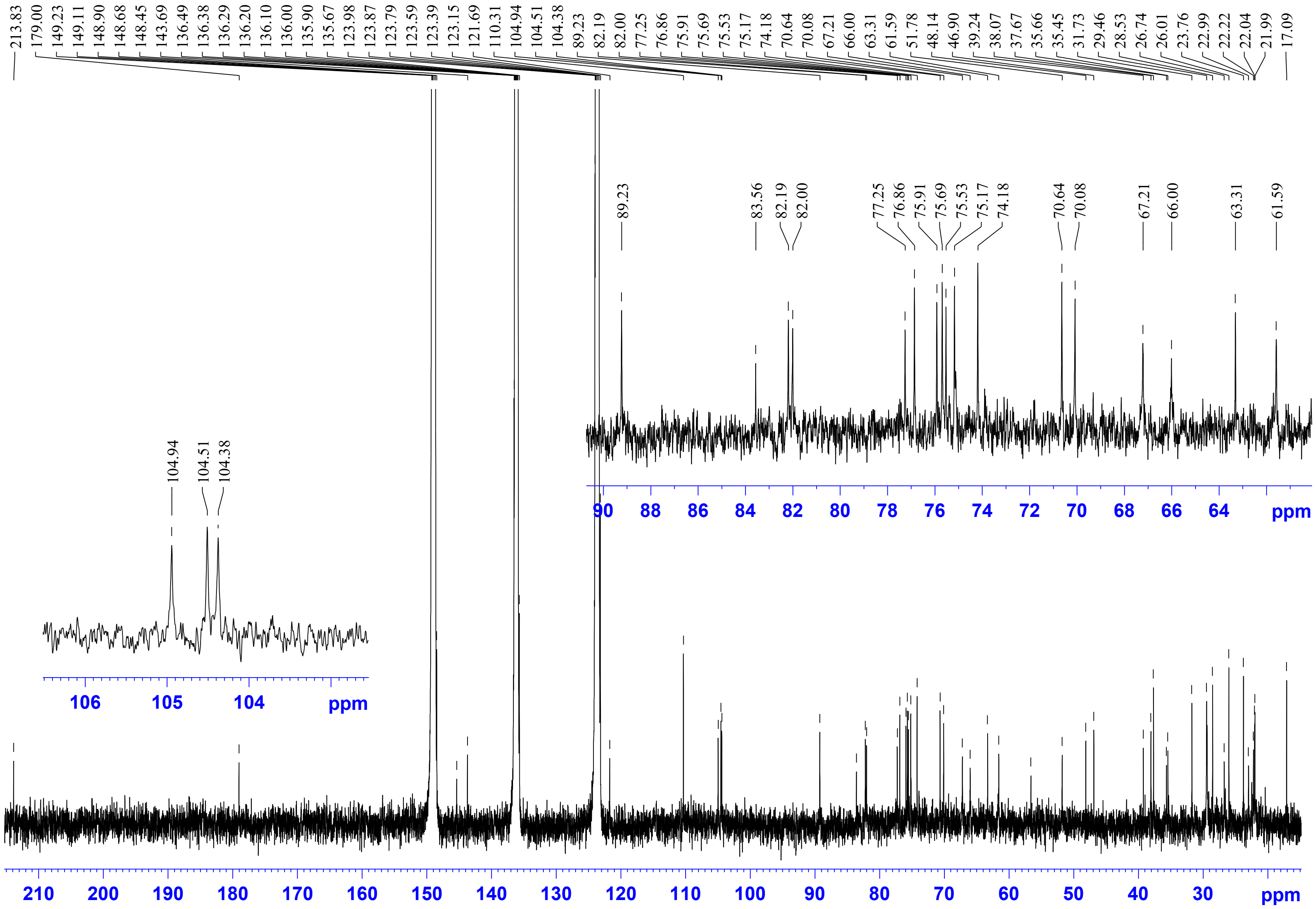

Fig. 33. The ^{13}C NMR (176.04 MHz) spectrum of psolusoside H (**5**) in C_5D_5N

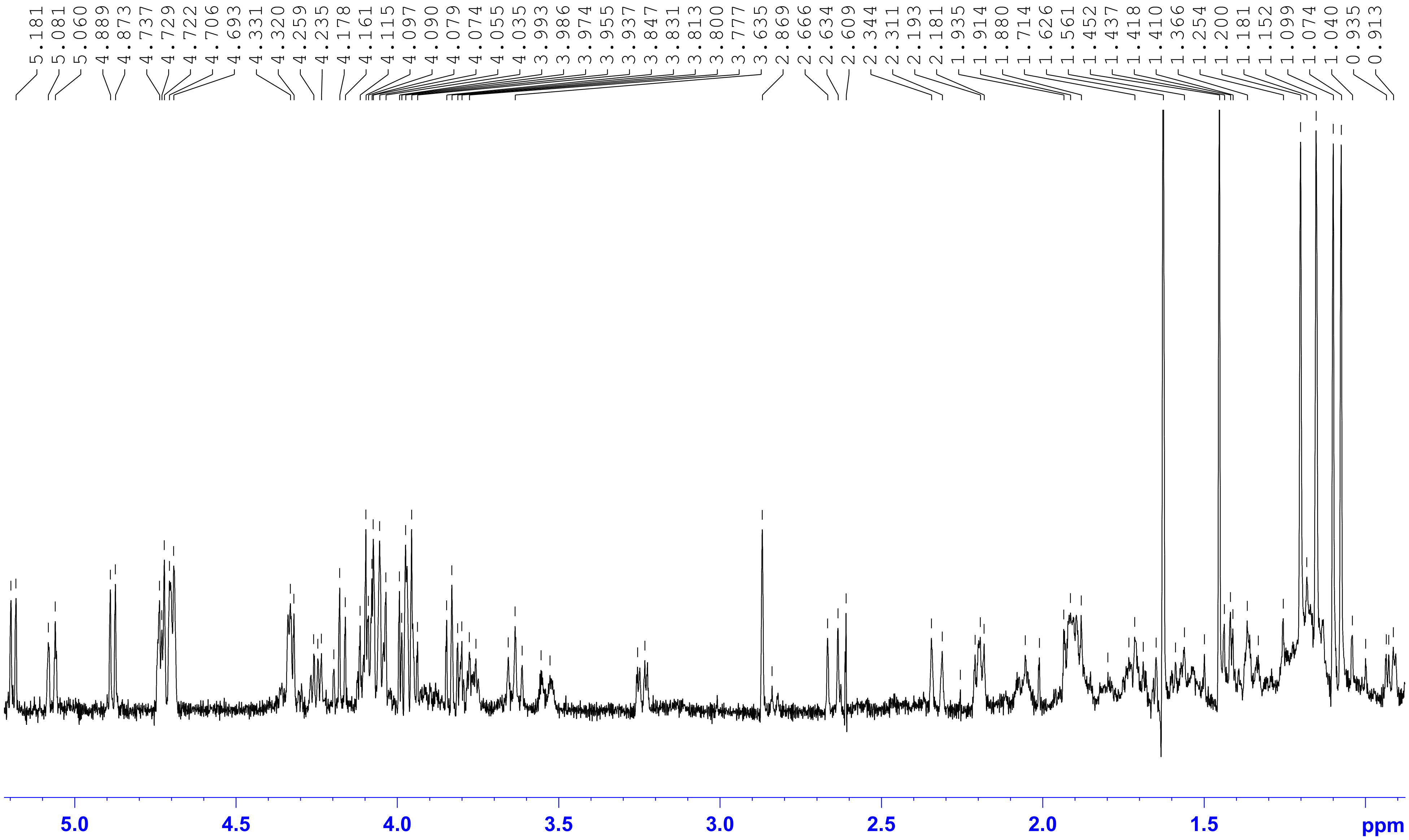

Fig. 34. The ^{1}H NMR (700.13 MHz) spectrum of psolusoside H (**5**) in C_5D_5N

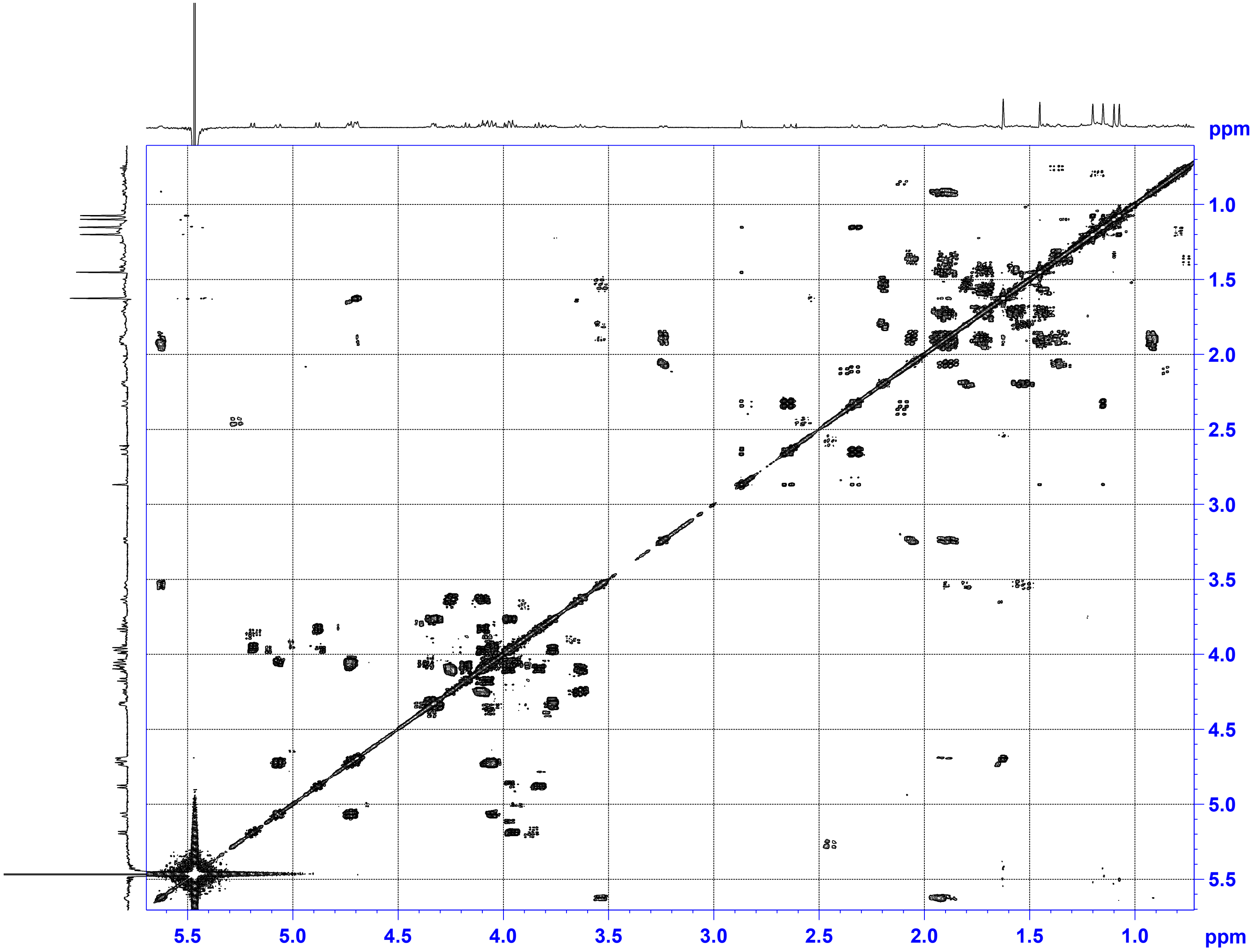

Fig. 35. The COSY (700.13 MHz) spectrum of psolusoside H (**5**) in C_5D_5N

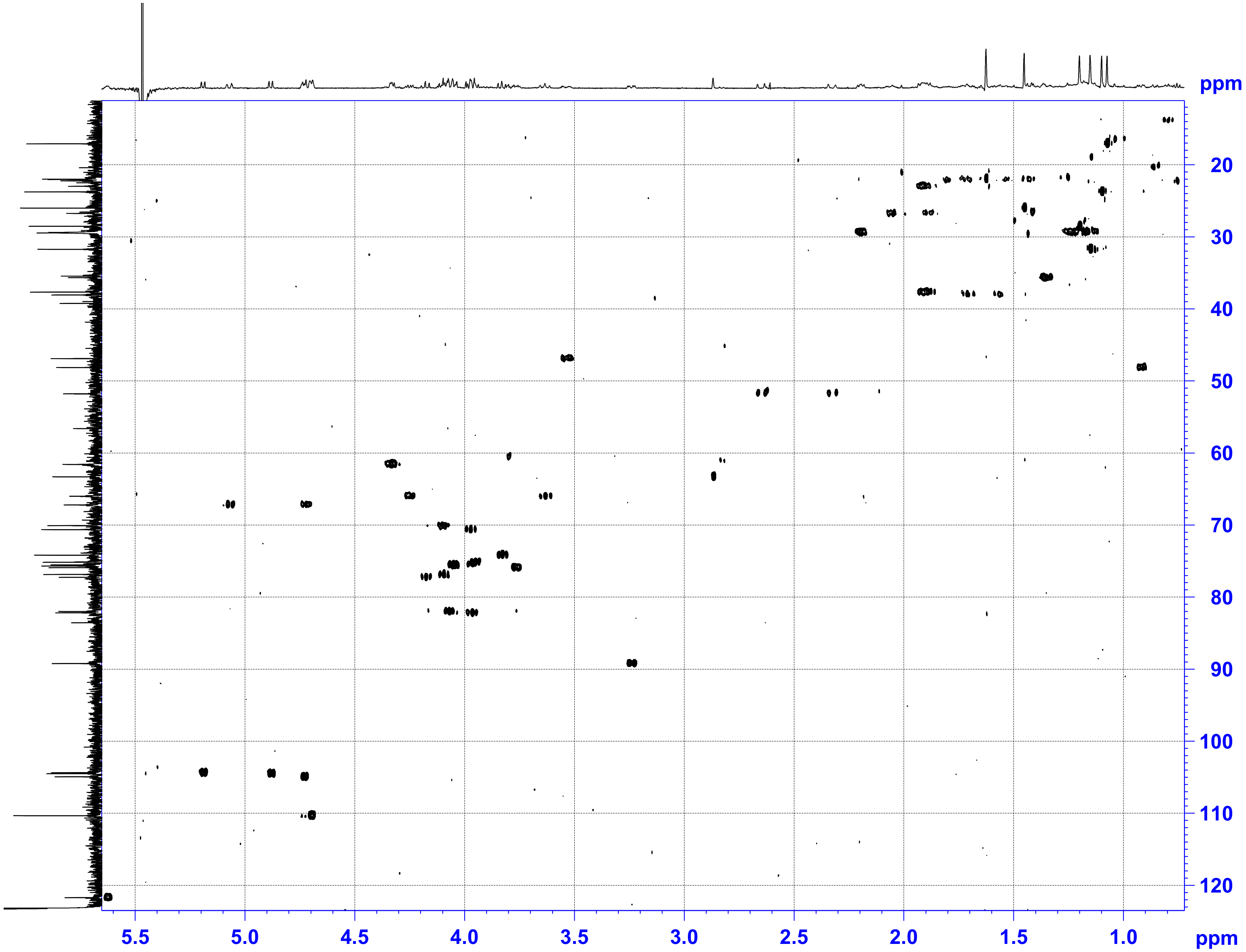

Fig. 36. The HSQC (700.13 MHz) spectrum of psolusoside H (**5**) in C_5D_5N

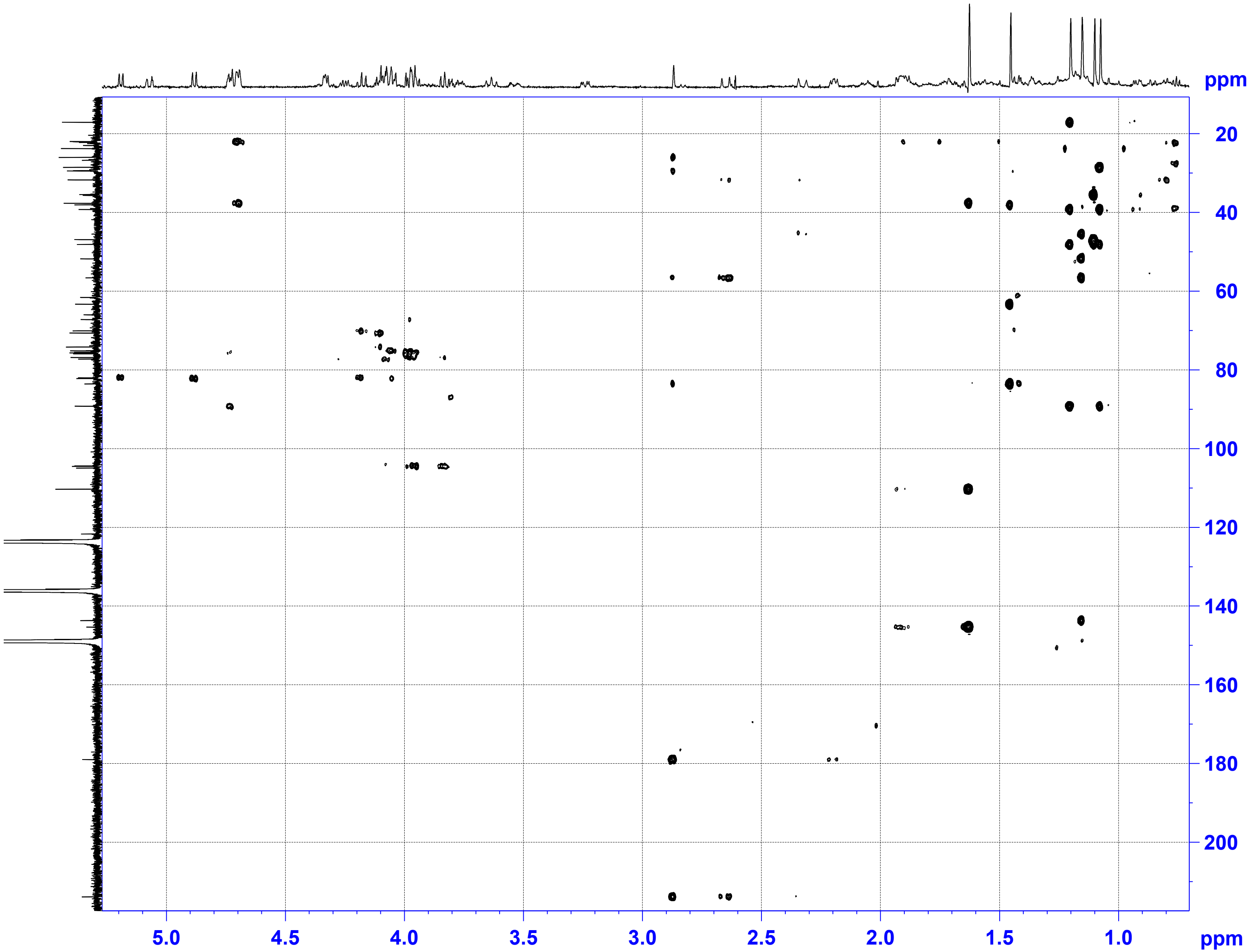

Fig. 37. The HMBC (700.13 MHz) spectrum of psolusoside H (**5**) in C_5D_5N

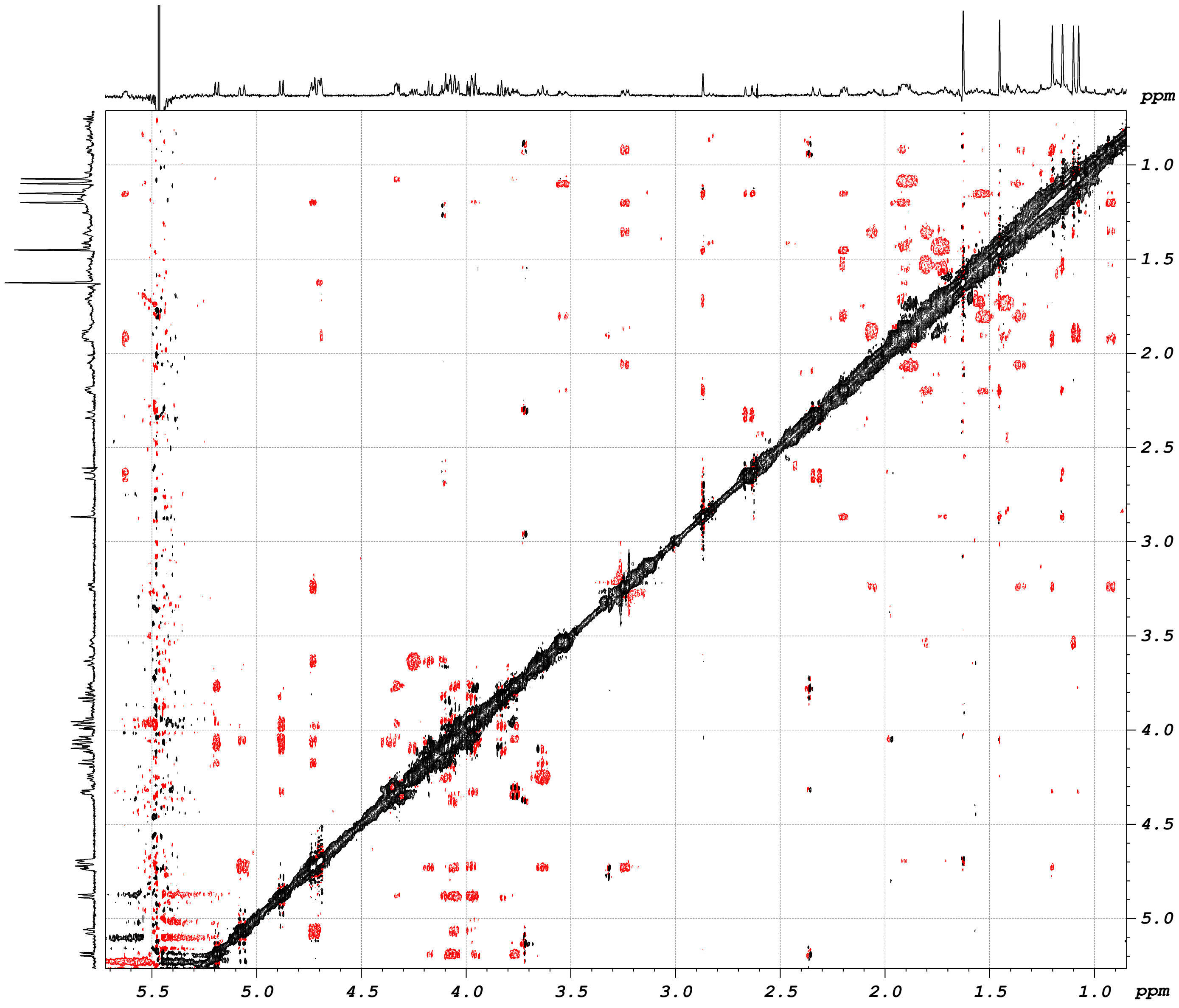

Fig. 38. The ROESY (500.13 MHz) spectrum of psolusoside H (**5**) in C_5D_5N

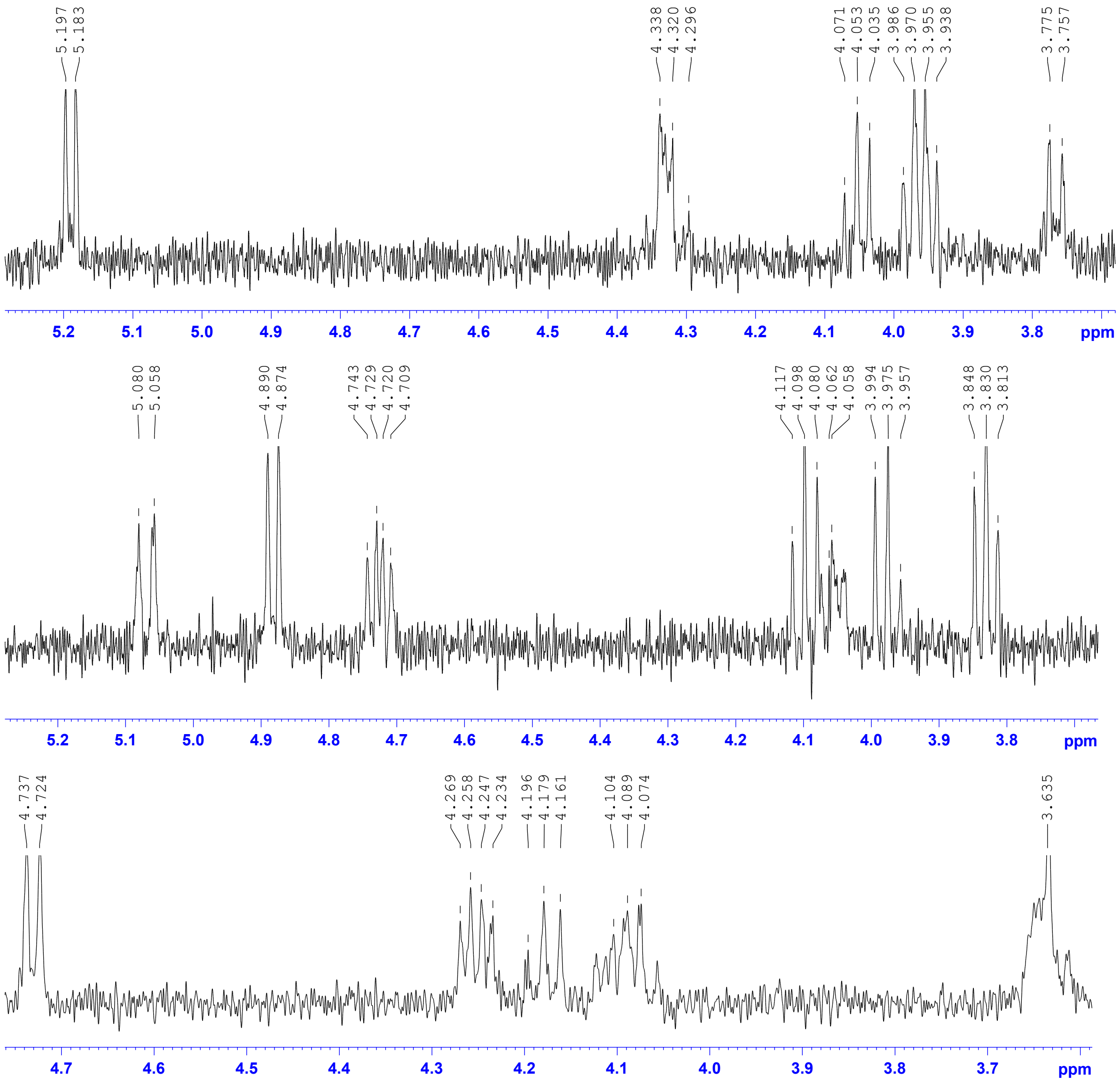

Fig. 39. 1D TOCSY (700.13 MHz) spectra of psolusosides H (**5**) and H1 (**6**) in C_5D_5N

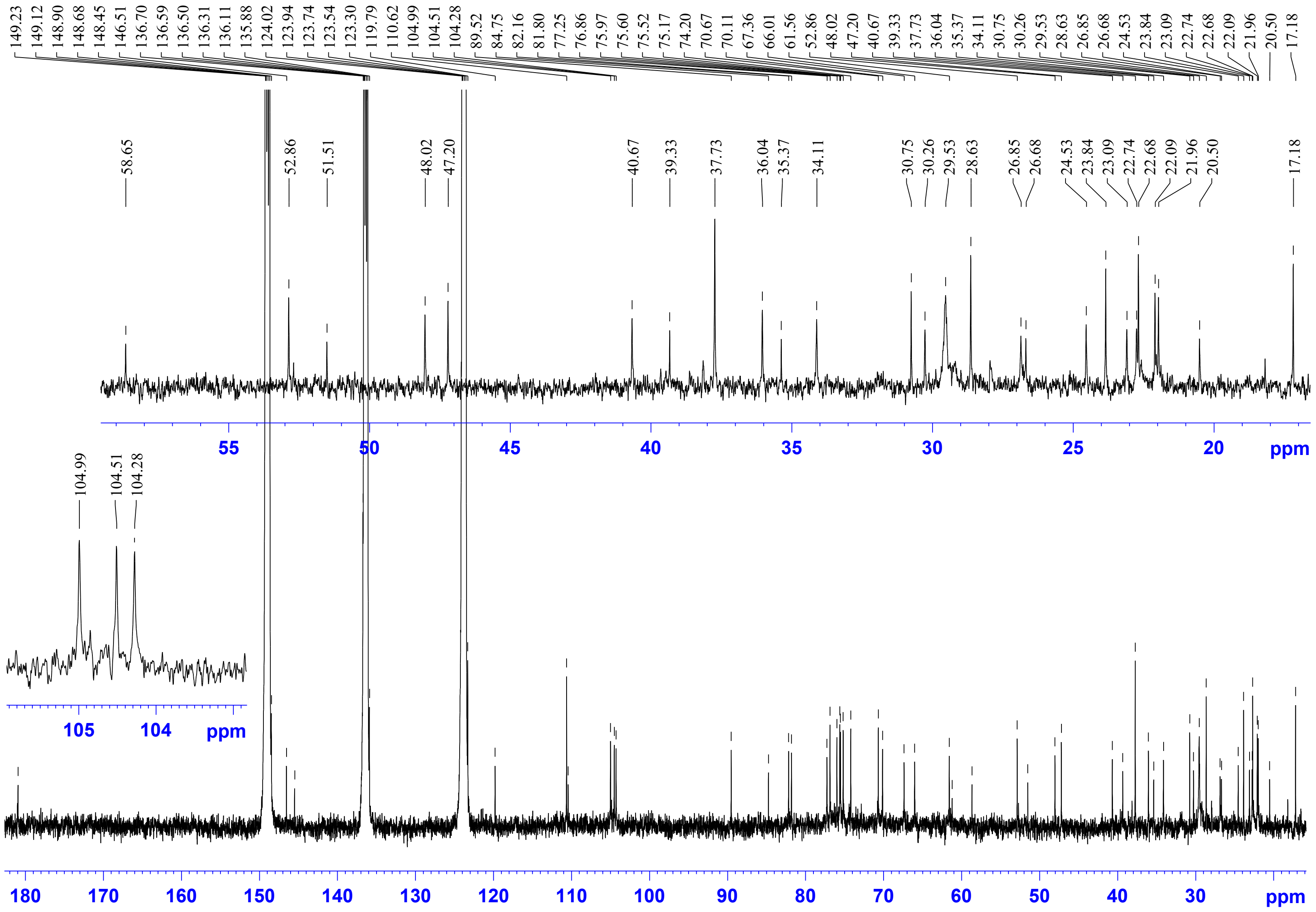

Fig. 40. The ^{13}C NMR (176.04 MHz) spectrum of psolusoside H_1 (**6**) in C_5D_5N

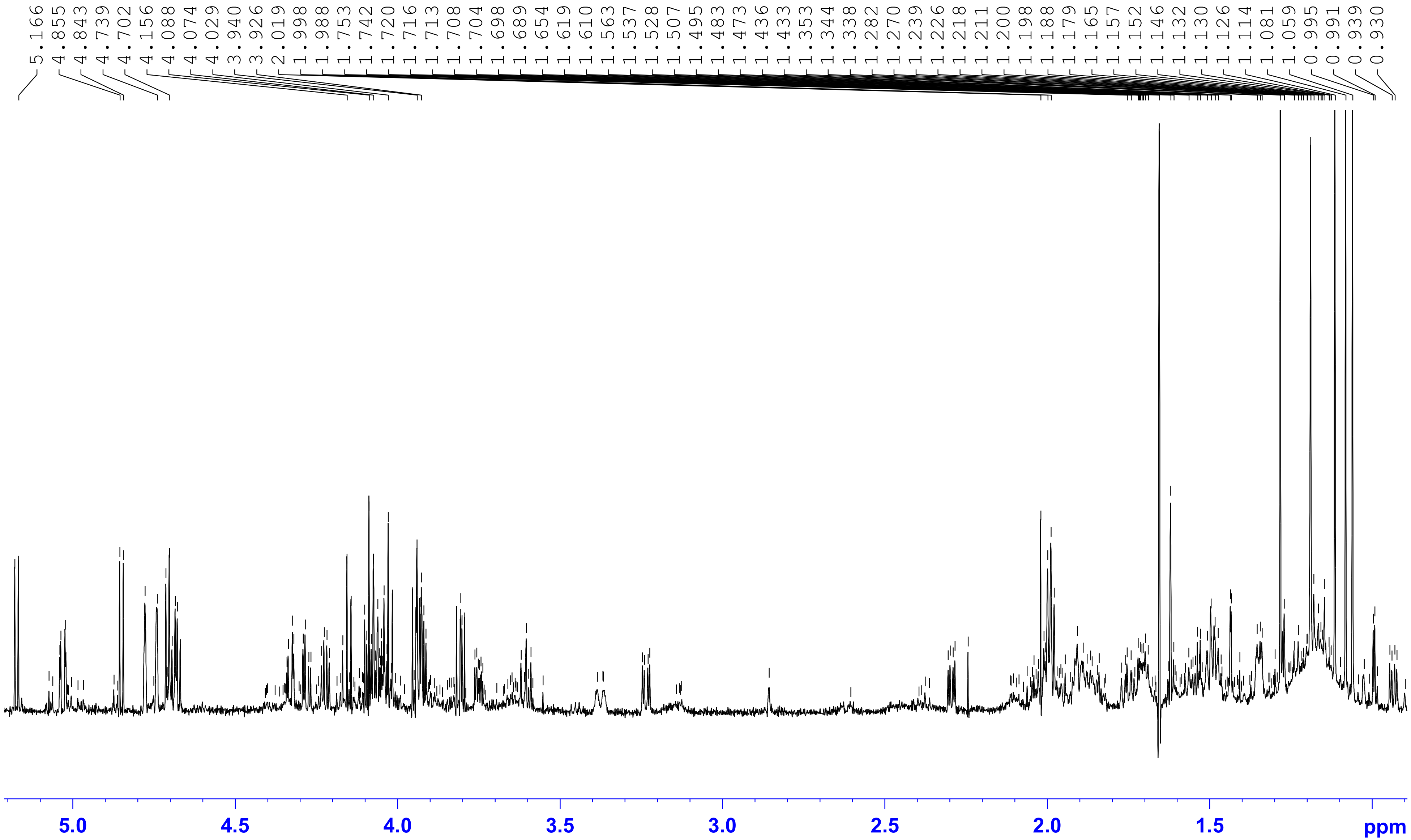

Fig. 41. The ^{1}H NMR (700.13 MHz) spectrum of psolusoside H_1 (**6**) in C_5D_5N

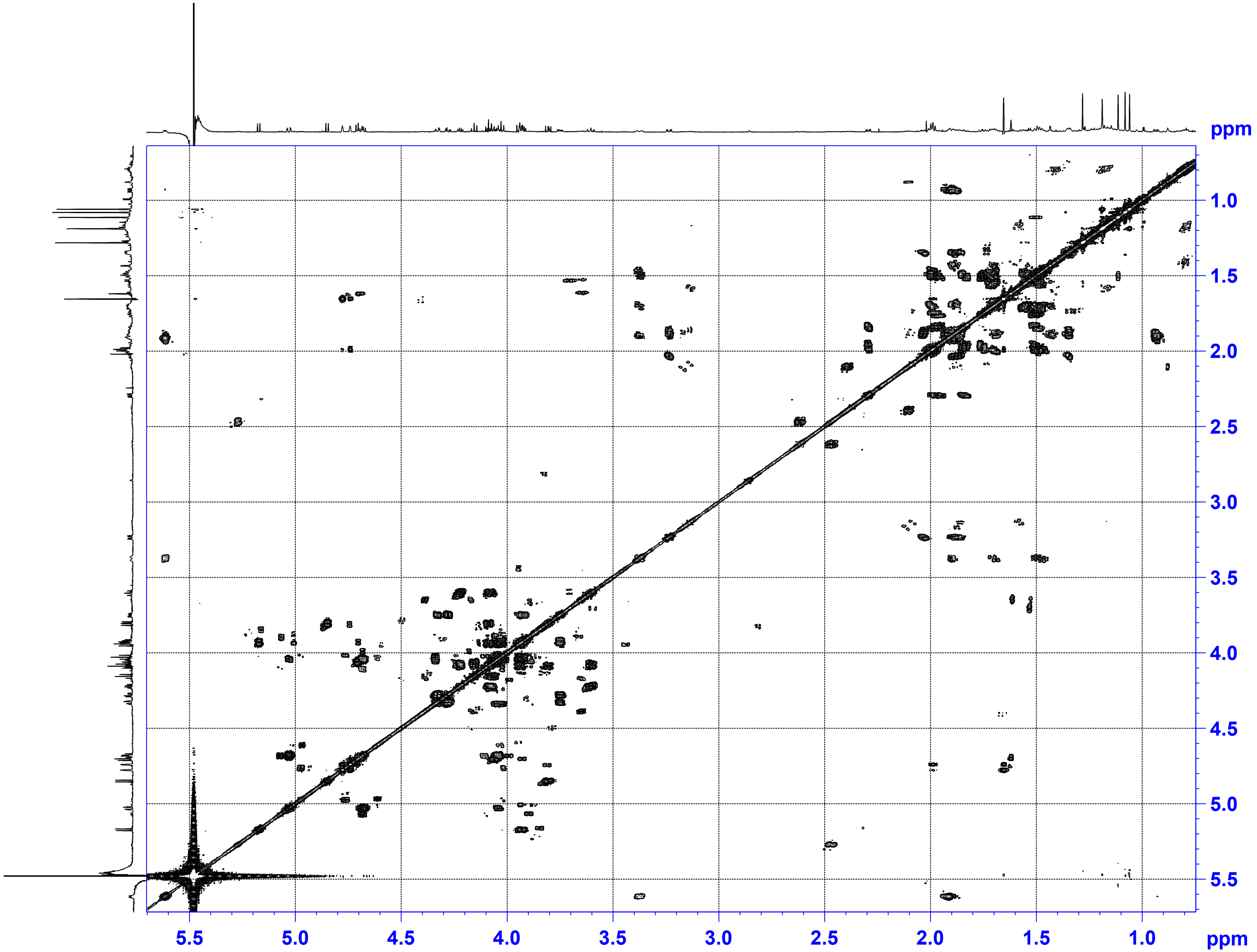

Fig. 42. The COSY (700.13 MHz) spectrum of psolusoside H_1 (**6**) in C_5D_5N

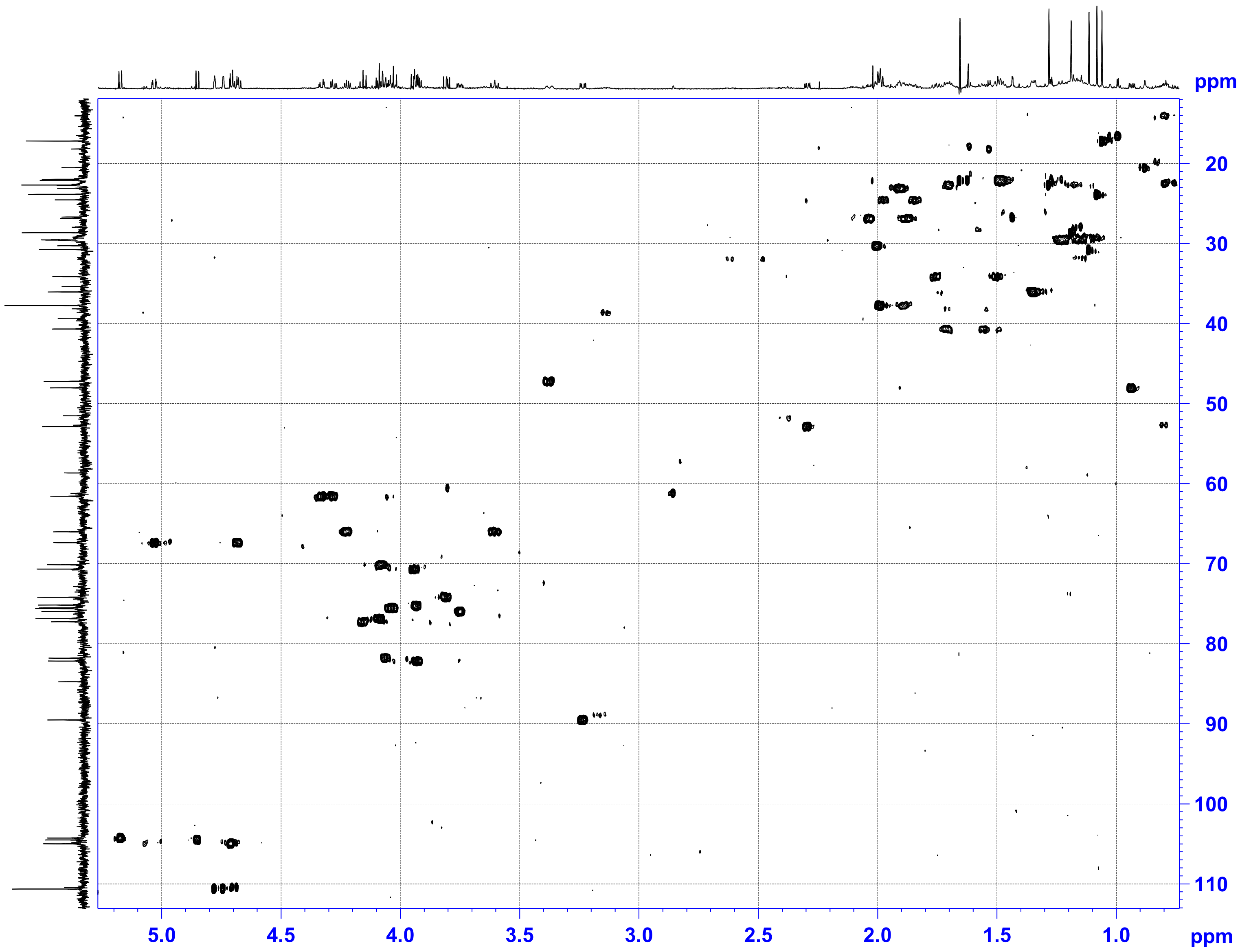

Fig. 43. The HSQC (700.13 MHz) spectrum of psolusoside H1 (**6**) in C_5D_5N

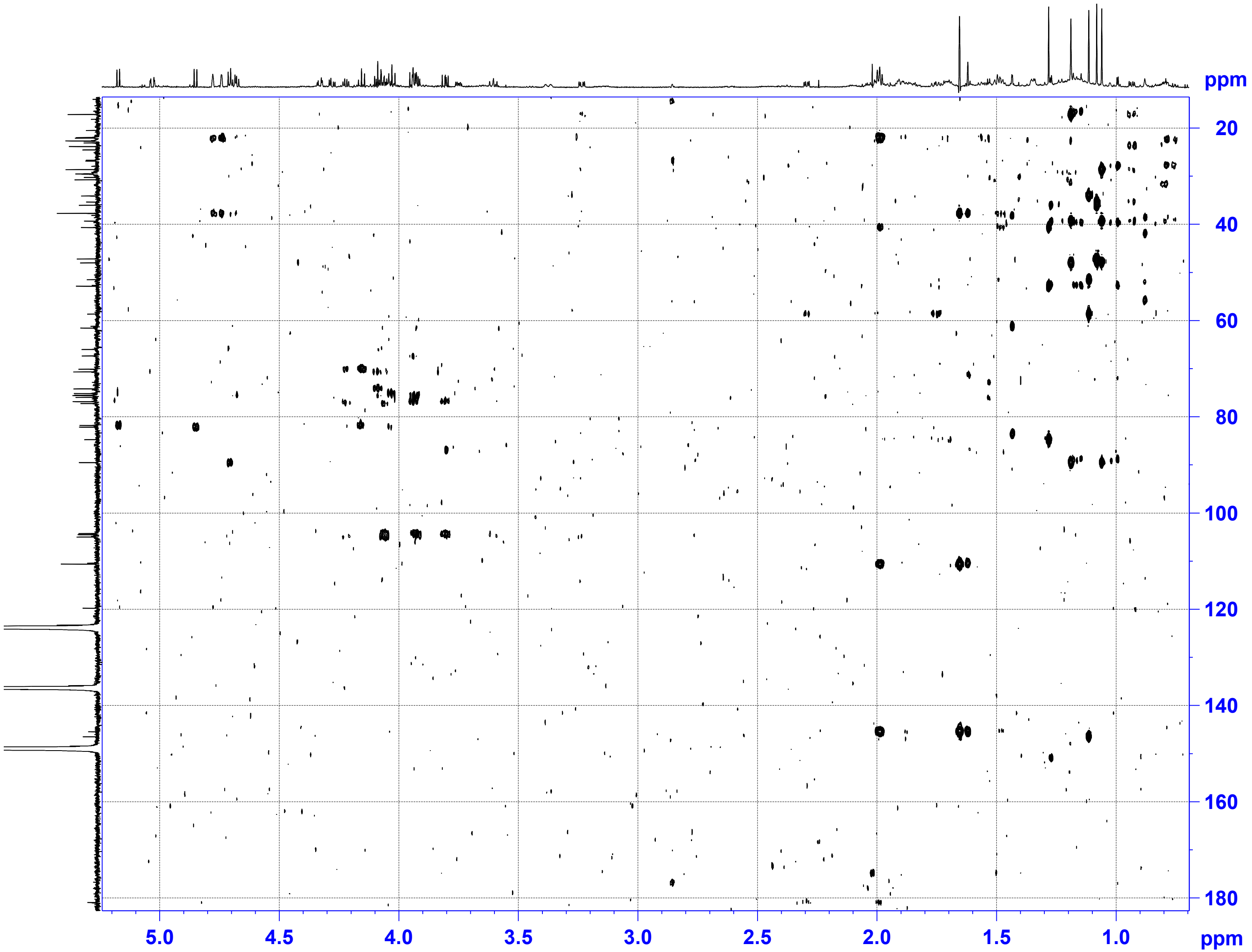

Fig. 44. The HMBC (700.13 MHz) spectrum of psolusoside H1 (**6**) in C_5D_5N

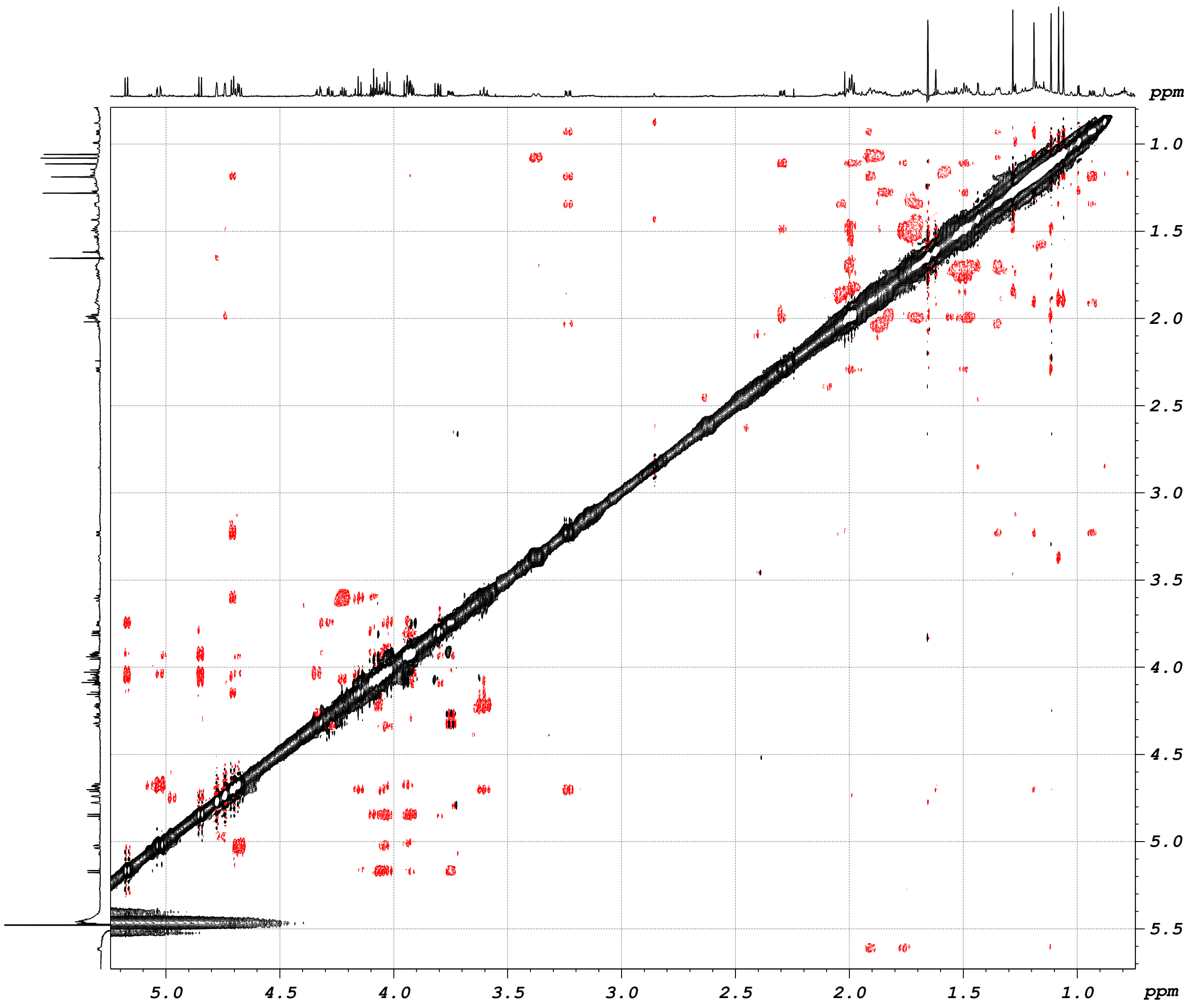

Fig. 45. The ROESY (500.13 MHz) spectrum of psolusoside H1 (**6**) in C_5D_5N

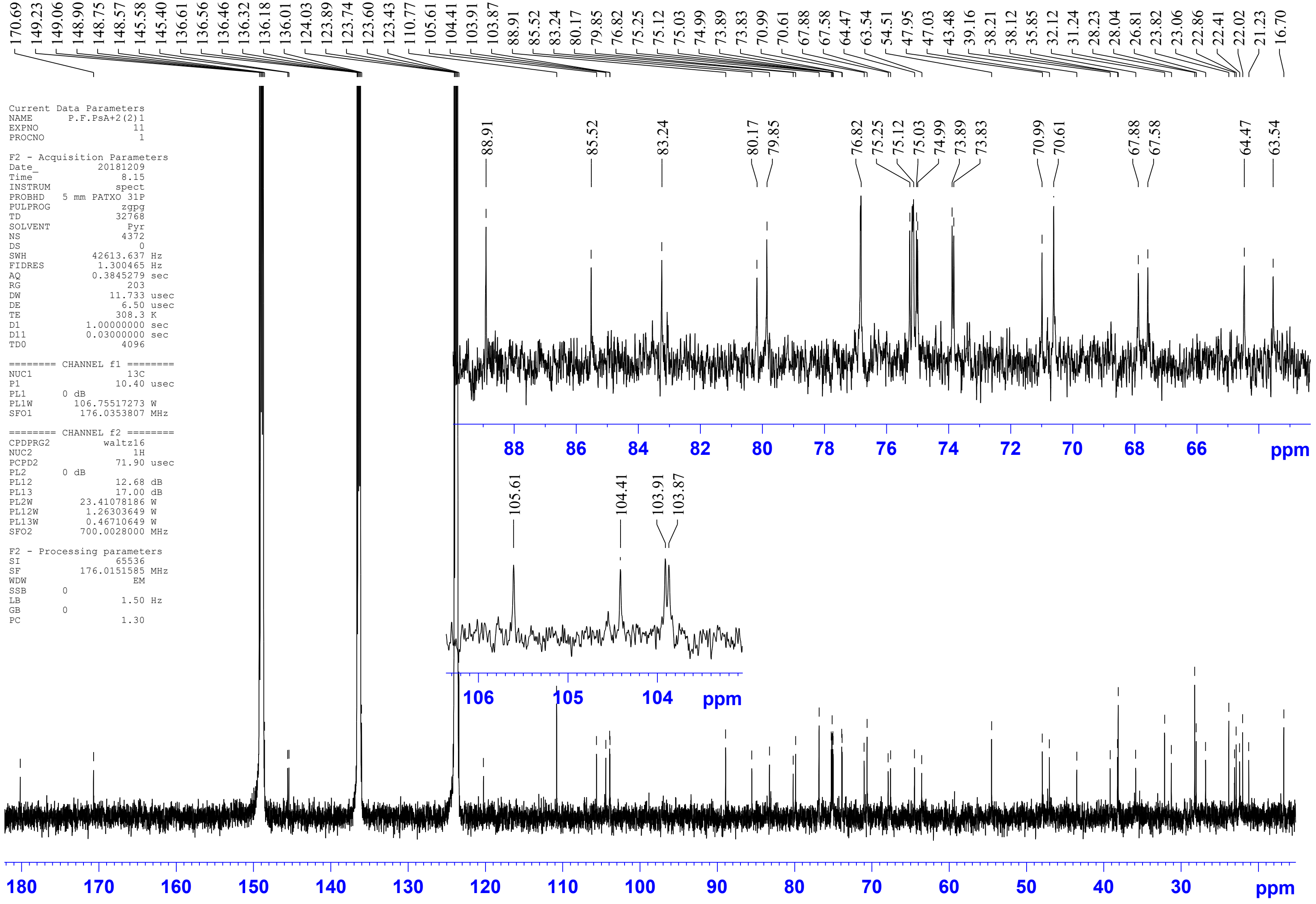

Fig. 46. The ^{13}C NMR (176.04 MHz) spectrum of psolusoside I (**7**) in C_5D_5N/D_2O (4/1)

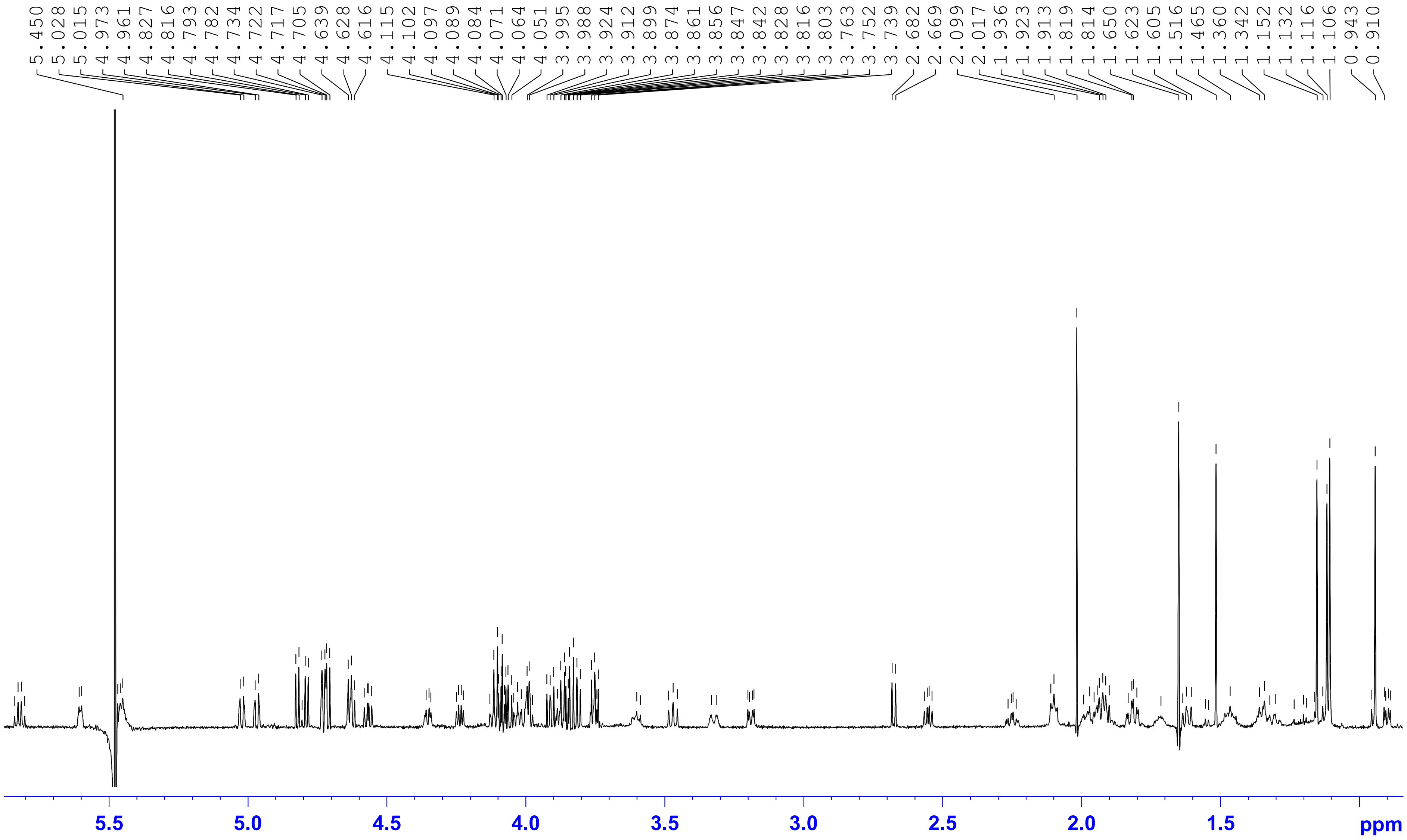

Fig. 47. The ^{1}H NMR (700.13 MHz) spectrum of psolusoside I (**7**) in C_5D_5N/D_2O (4/1)

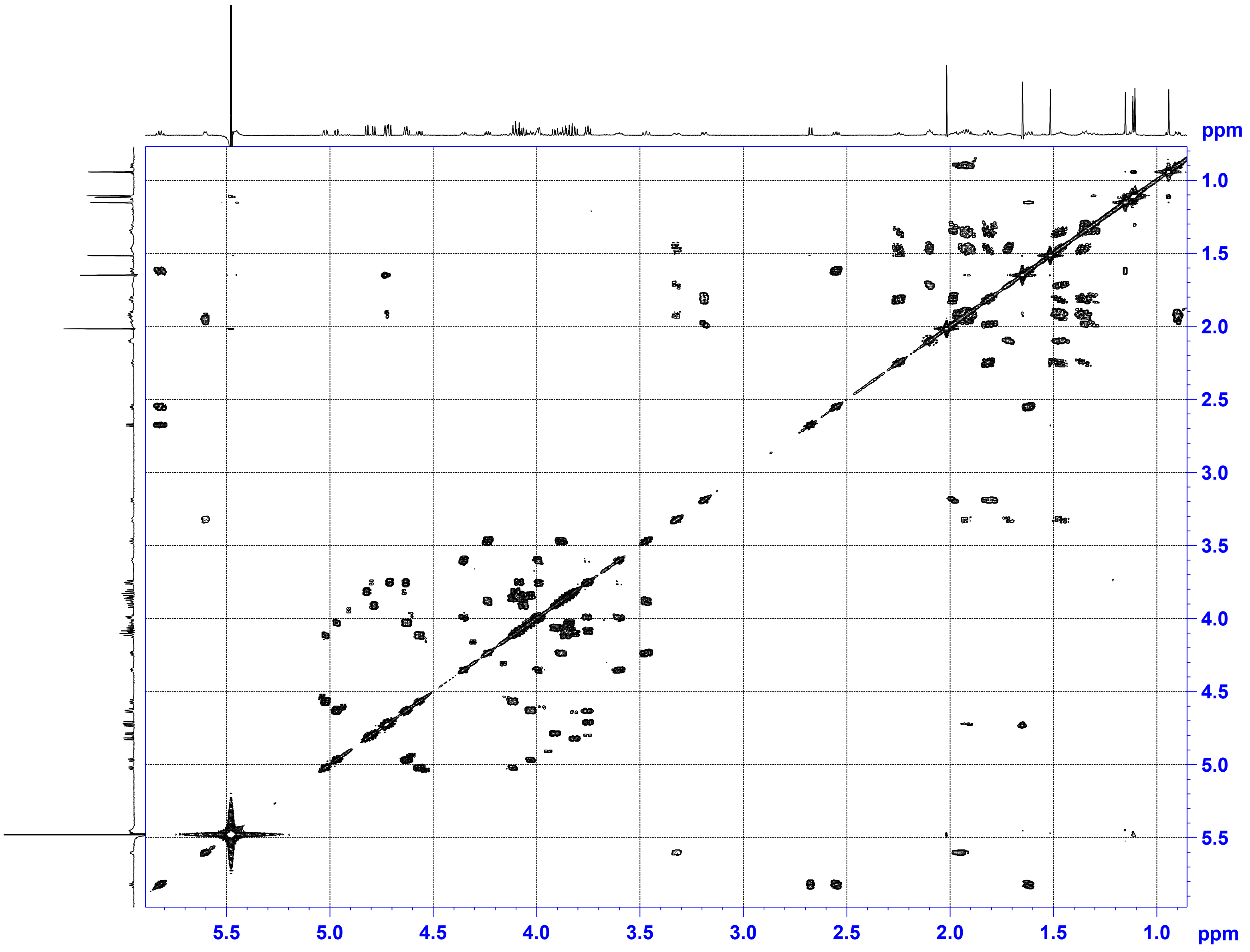

Fig. 49. The COSY (700.13 MHz) spectrum of psolusoside I (**7**) in C_5D_5N/D_2O (4/1)

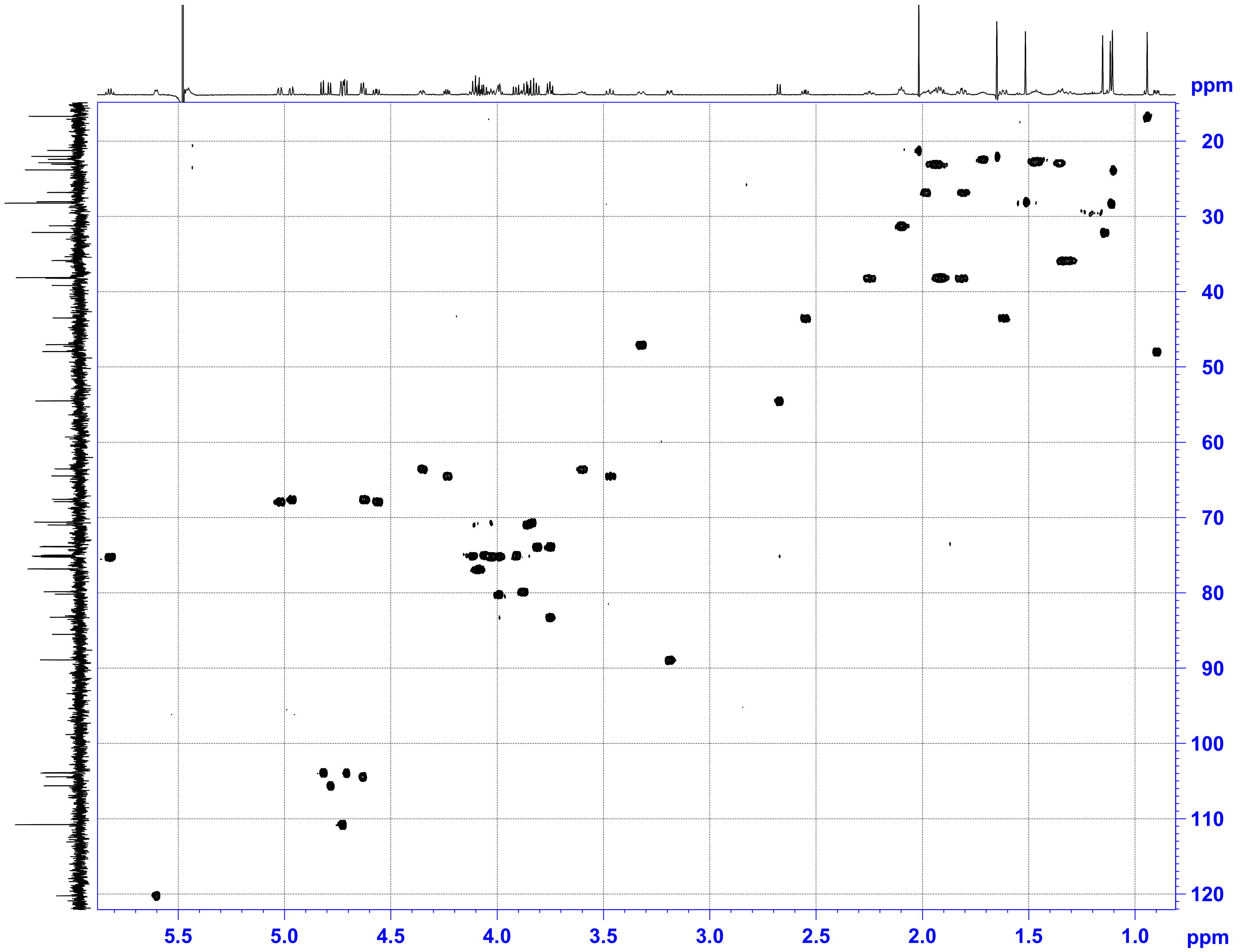

Fig. 50. The HSQC (700.13 MHz) spectrum of psolusoside I (**7**) in C_5D_5N/D_2O (4/1)

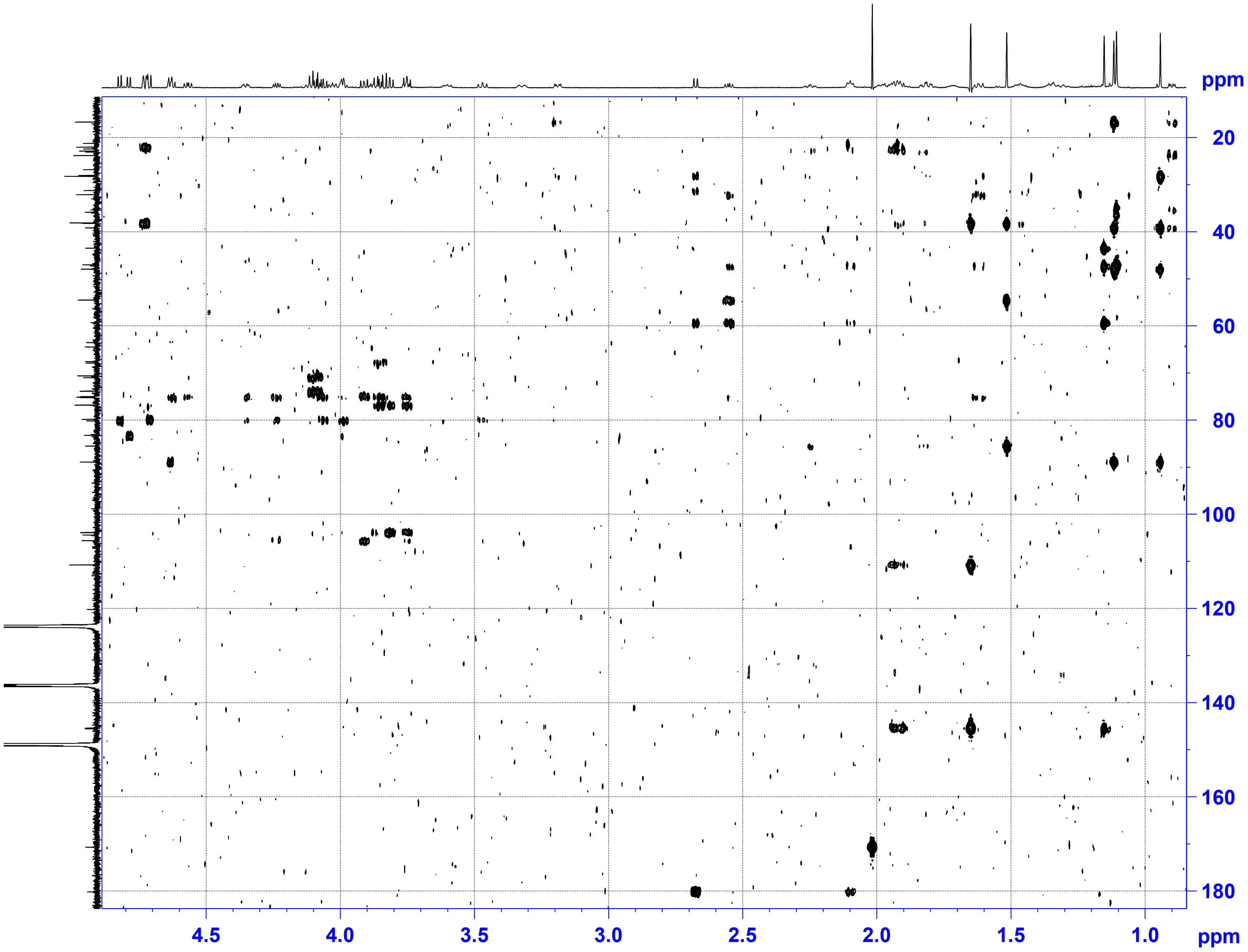

Fig. 51. The HMBC (700.13 MHz) spectrum of psolusoside I (**7**) in C_5D_5N/D_2O (4/1)

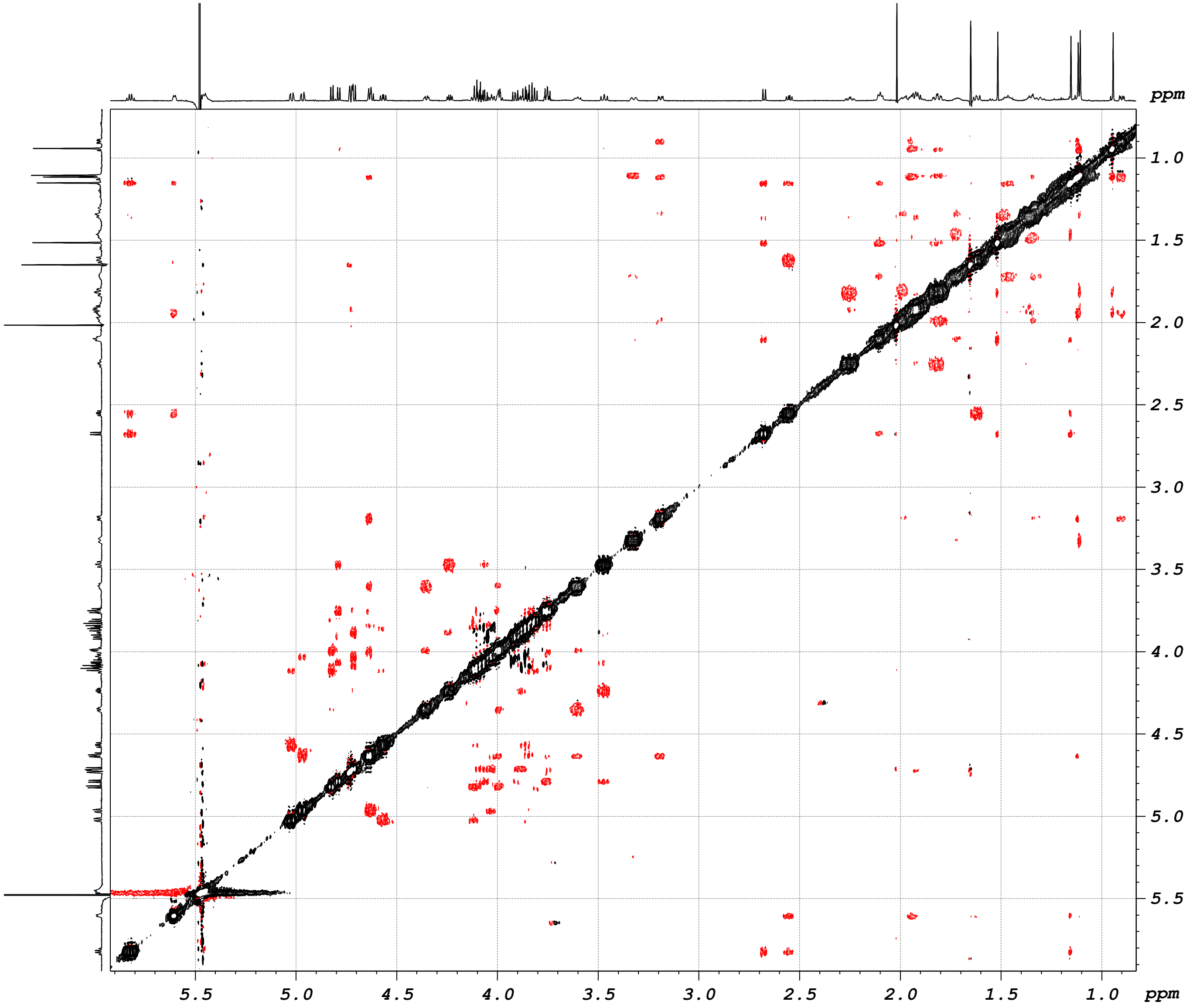

Fig. 52. The ROESY (500.13 MHz) spectrum of psolusoside I (**7**) in C_5D_5N/D_2O (4/1)

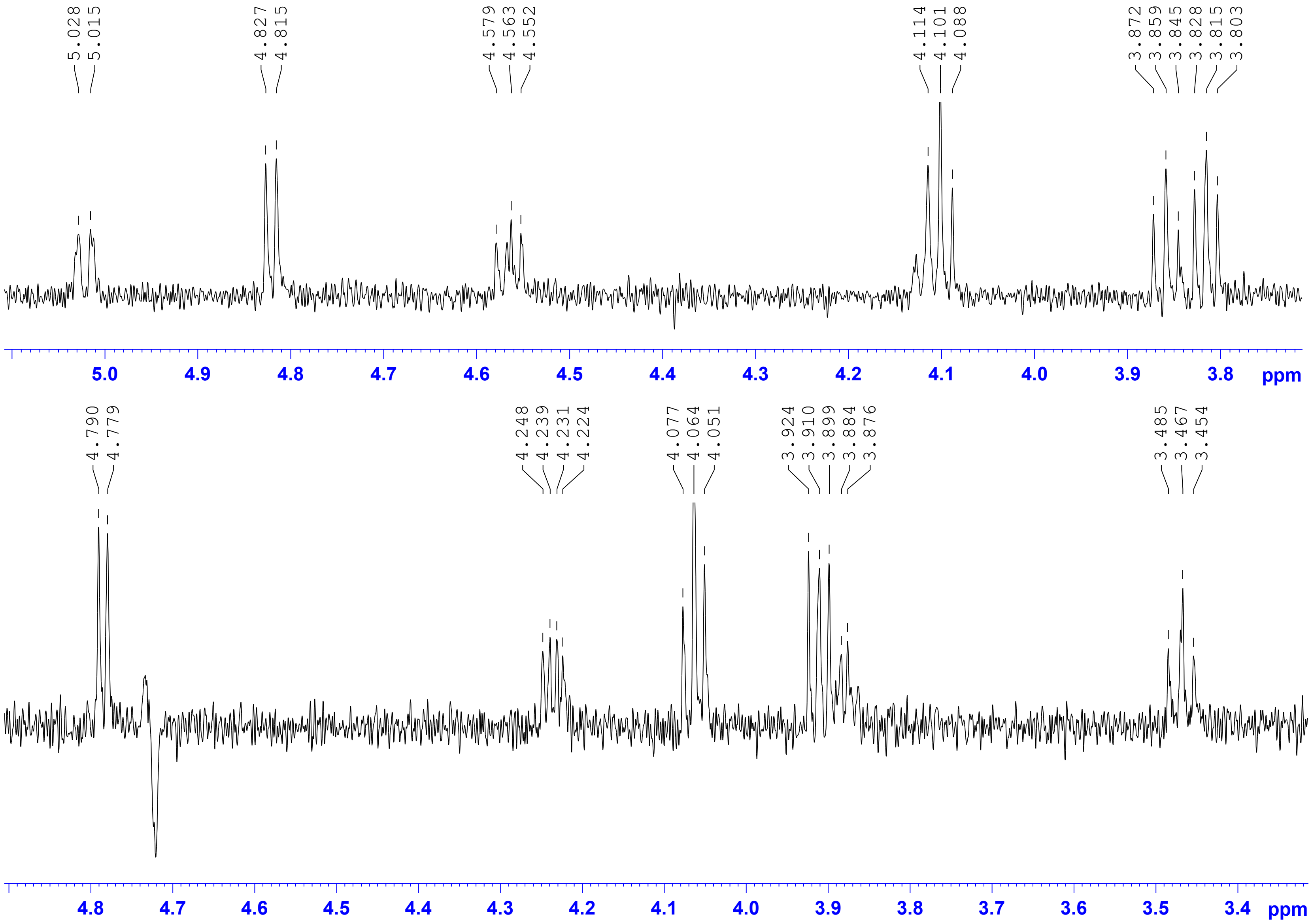

Fig. 53. 1D TOCSY (700.13 MHz) spectra of psolusoside I (**7**) in C_5D_5N/D_2O (4/1)

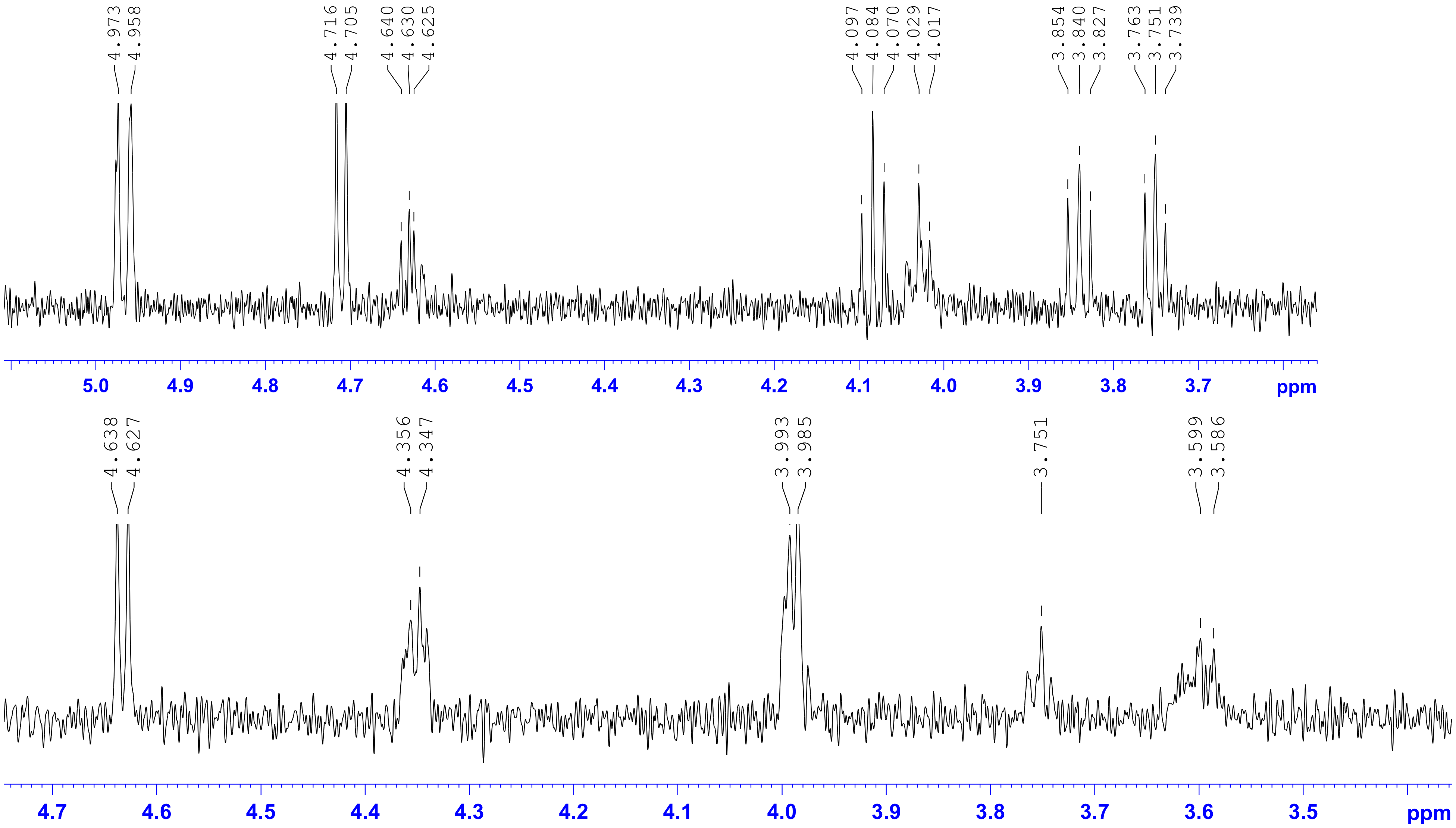

Fig. 54. 1D TOCSY (700.13 MHz) spectra of psolusoside I (**7**) in C_5D_5N/D_2O (4/1)
